# Ancestral [Fe-S] biogenesis system SMS has a unique mechanism of cluster assembly and sulfur utilization

**DOI:** 10.1371/journal.pbio.3003223

**Published:** 2025-06-25

**Authors:** Macha Dussouchaud, Markel Martinez-Carranza, Pierre-Simon Garcia, Martin Clémancey, Geneviève Blondin, Jean Michel Betton, Ahmed Haouz, Simonetta Gribaldo, Sandrine Ollagnier de Choudens, Ludovic Sauguet, Ariel Mechaly, Frédéric Barras

**Affiliations:** 1 Department of Microbiology, Unit Stress Adaptation and Metabolism in Enterobacteria, Institut Pasteur, Université Paris Cité, UMR CNRS 6047, Paris, France; 2 Department of Structural Biology, Unit Architecture and Dynamics of Biological Macromolecules, Institut Pasteur, Université Paris Cité, UMR CNRS 3528, Paris, France; 3 Department of Microbiology, Unit Evolutionary Biology of the Microbial Cell, Institut Pasteur, Université Paris Cité, Paris, France; 4 Université Grenoble Alpes, CNRS, CEA, IRIG, Laboratoire de Chimie et Biologie des Métaux, Grenoble, France; 5 Center for Technological Resources and Research, Institut Pasteur, Université Paris Cité, UMR CNRS 3528, Paris, France; Rutgers University-Robert Wood Johnson Medical School, UNITED STATES OF AMERICA

## Abstract

[Fe-S] clusters are ancient and ubiquitous protein co-factors, which contributed to the emergence of life in an anoxic planet. We have recently identified two minimal [Fe-S] biogenesis systems, MIS and SMS, inferred to be ancestral systems dating back to the Last Universal Common Ancestor and which gave rise to the well-studied modern Iron-Sulfur Cluster (ISC), Nitrogen Fixation (NIF), and Sulfur Mobilization (SUF) machineries. The present study focuses on the ancestor SMS from the hyperthermophilic archaeon *Methanocaldococcus jannaschii*. Biochemical and structural studies showed that SMS is made of a SmsC_2_B_2_ heterotetratmer wherein the SmsC subunit hosts both ATP and [Fe-S] cluster binding sites. Binding of ATP and assembly of [Fe-S] were found to be mutually exclusive allowing for a regulatory coupling between binding of both substrates. Mutagenesis and in vitro transfer experiments revealed the key role of SmsC-contained Cys residues in cluster assembly. Strikingly, the SMS system rescued a non-viable *Escherichia coli* strain lacking endogenous ISC and SUF systems grown under anoxic conditions, in the presence of Na_2_S, indicating that sulfide is a source of sulfur for SMS. In addition, we predict that most archaea SmsC proteins hold a similar C-terminal [Fe-S] cluster assembly site. Taking into account those unique structural and functional features, we propose a mechanistic model describing how SmsC_2_B_2_ assembles and distributes [4Fe-4S] clusters. Altogether this study established SMS as a new *bona fide* [Fe-S] biogenesis system that operated in anaerobic prokaryotes prior to evolve to SUF after the Great Oxydation Event.

## Abstract 2

The minimal [Fe-S] biogenesis systems, MIS and SMS, are thought to be ancestral to modern ISC, NIF, and SUF machineries. This study shows that the ancestral SMS system from Methanocaldococcus jannaschii assembles [Fe-S] clusters through unique ATP-regulated mechanisms and uses sulfide as a sulfur source.

## Introduction

Iron-sulfur ([Fe-S]) clusters are inorganic entities that contributed to early stages and subsequent evolution of life [1–4]. Life emerged in an anoxic, iron- and sulfur-rich environment. Minerals containing [Fe-S] clusters formed spontaneously under such conditions, such as pyrite (FeS_2_) or mackinawite (FeS). Very likely, these abiotically produced [Fe-S] clusters contributed to early forms of life and their subsequent evolution by providing nascent living systems with redox power [5,6]. Subsequently, the Great Oxidation Event (GOE) led to increased oxygenation of the atmosphere and limitation of soluble bioavailable iron. In the late 90s, the discovery of ISC, SUF, and NIF machineries led to the widely assumed notion that [Fe-S] biogenesis ought to be catalyzed to mitigate the GOE-caused deleterious conditions [7–12]. However, our recent discovery of Minimal ISC System (MIS) and Suf Minimal System (SMS) and their inference in the Last Universal Common Ancestor (LUCA) suggested the necessity to catalyze biotically [Fe-S] biogenesis even under favorable pre-GOE conditions [13,14]. This discovery raised exciting questions about the functioning of the ancestral systems, SMS and MIS, and their subsequent evolution to give rise to SUF and ISC/NIF, respectively.

In present-day organisms, [Fe-S] clusters are essential cofactors of proteins, controlling multiple essential cellular processes such as DNA replication and repair, protein synthesis, central metabolism, photosynthesis, respiration, and antiviral defenses. The three machineries, ISC, SUF, and NIF have been the focus of multiple studies in both Prokaryotes and Eukaryotes [7–12]. Briefly, these machineries include a cysteine desulfurase, which provides sulfur from l-cysteine, to a scaffold component that assembles the [Fe-S] cluster, which is subsequently delivered to cellular recipient proteins via dedicated [Fe-S] carriers. The MIS system follows these rules as it has a cysteine desulfurase and a scaffold, but no carrier. The simplicity of the SMS raises additional questions, which are discussed below.

SMS is the ancestor of SUF and has been retained mostly in Archaea. With respect to the six components of the SUF system, SMS has only two components, SmsC and SmsB, encoded by the *smsCB* operon. We previously showed in vitro that SmsCB proteins bind a [Fe-S] cluster, which can be transfered to apo-aconitase, suggesting that SmsCB acts as a scaffold [14]. However, in the absence of a cysteine desulfurase and carrier, it remains unknown how SmsCB makes and distributes [Fe-S] clusters, and what is the source of sulfur. Here, we have carried out a multidisciplinary analysis of the SMS system from *Methanocaldococcus jannaschii,* a hyperthermophilic methanogenic archaeon. By combining X-ray crystallography and cryogenic electron microscopy (cryo-EM), we determined the structure of the *M. jannaschii* [4Fe-4S]-bound SmsC_2_B_2_ complex. Unexpectedly, the [4Fe-4S] cluster binding site was located in only one of the SmsC subunits at a C-terminal flexible loop region that is disordered in its absence. Mutagenesis and in vitro [Fe-S] cluster transfer experiments revealed the key role of C-terminal located Cys residues in building the cluster. Biochemical analysis revealed a mutual exclusive relationship between ATP binding and [Fe-S] assembly, leading credence to a regulatory interplay within the interaction of SmsC and both of its substrates. Last, in vivo genetic analysis strongly suggested that mineral sulfide acts as a source of sulfur for SMS to assemble [Fe-S] clusters. Our results demonstrated the uniqueness of the SMS machinery and endowed it with a status of new *bona fide* [Fe-S] biogenesis system. A wide arrays of archaea, including methanogens and gut inhabitants, is relying on SMS to multiply and this study will undoubtedly help in our understanding of these microbial communities.

## Results

### SmsB and SmsC form an SmsC_2_B_2_ hetero-tetramer complex that assembles a [4Fe-4S] cluster

**Fig 1:**
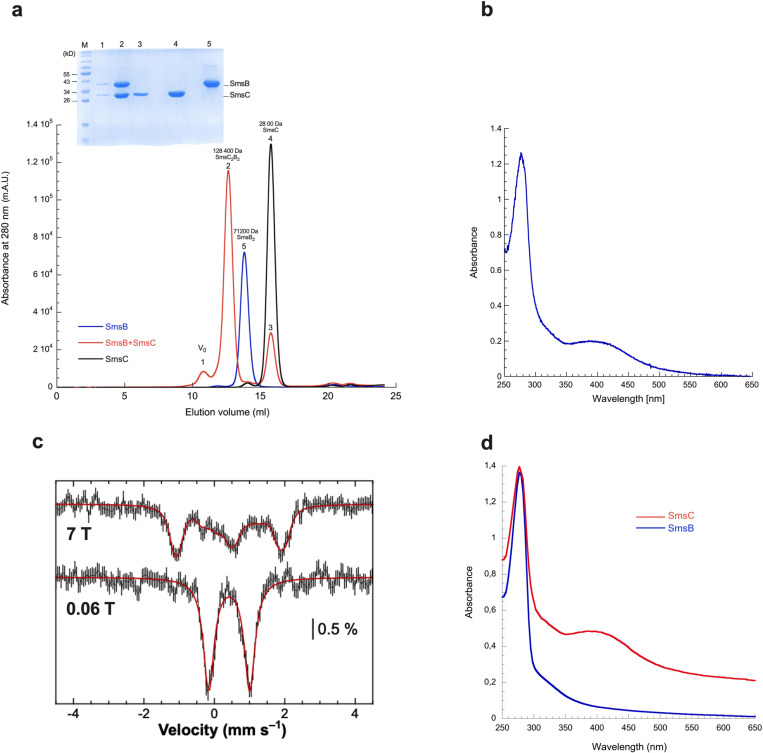
Spectroscopy analysis of SmsC_2_B_2_ complex. **(a)** SDS-PAGE and size exclusion chromatography profile of purified Sms proteins. Lane (1): V_0_, lane (2): SmsC_2_B_2_, lane (3): SmsC from the SmsC_2_B_2_, lane (4): SmsC monomers, lane (5): SmsB homodimers. **(b)** UV-Vis absorption spectrum of SmsC_2_B_2_. SmsC_2_B_2_ (38.5 μM) was incubated with 5 equivalents of Fe^2+^/SmsC_2_B_2_, 5 equivalents of Na_2_S/SmsC_2_B_2_, and 3 mM DTT**. (c)** 6 K Mössbauer spectra (black vertical bars) of SmsC_2_B_2_ (350 µM, 3.6 Fe and 3.4 S/SmsC_2_B_2_) recorded using a 0.06 T and a 7 T external magnetic field applied parallel to the γ-beam. The simulations assuming a unique iron site are overlaid as thick red solid lines (see text for parameters). **(d)** UV-Vis absorption spectrum of chemically reconstituted Sms proteins. SmsC (in red) (70 μM) and SmsB (in blue) (62 μM) were incubated with 5 equivalents of Fe^2+^/SmsC_2_ or SmsB_2_, 5 equivalents of Na_2_S/SmsC_2_ or SmsB_2_ and 3 mM DTT. The data underlying this figure can be found in Fig 1 and S1 Data.

Biochemical characterization of the SmsCB protein was carried out. To purify the SmsCB protein complex, we independently overproduced recombinant His-tagged SmsC and SmsB proteins in *Escherichia coli* and reassembled the complex in vitro after Ni affinity chromatography and peptide tag removal. A homogenous preparation of the SmsCB complex was then purified by size exclusion chromatography (Fig 1a). Purified SmsC, SmsB, and SmsCB eluted, on a calibrated size exclusion chromatography column, at a volume corresponding to a monomer (SmsC), a homodimer (SmsB_2_), and a hetero-tetramer (SmsC_2_B_2_), respectively (Fig 1a). That the purified SmsC_2_B_2_ was under its apo form was shown by a lack of absorption peak in the UV-visible spectrum, corresponding to [Fe-S] cluster, ATP or FAD. It was subsequently transferred to an anoxic chamber and chemical reconstitution of the [Fe-S] bound species (holo-form) was performed using a five-molar excess of iron and sulfur per SmsC_2_B_2_. Consistent with our previous report [14], UV-visible spectra of reconstituted complexes displayed an absorption band at 420 nm indicative of the presence of a [4Fe-4S] cluster on the SmsC_2_B_2_ complex (Fig 1b). Subsequent quantitation revealed that iron and sulfur content per mol of complex were 2.5 ± 0.1 Fe and 2.4 ± 0.15 S per chemically reconstituted SmsC_2_B_2_ complex. These results were consistent with the binding of one [4Fe-4S] cluster per SmsC_2_B_2_ complex. Increasing the molar excess of iron and sulfur led to the appearance of more metallic aggregates on the complex, but not binding of another cluster.

Mössbauer spectra of SmsC_2_B_2_, recorded at 5.8 K using a 0.06 and a 7 T external magnetic field applied along the *γ*-rays direction (Fig 1c), were strongly reminiscent of those previously reported for diamagnetic [4Fe-4S] clusters [15]. They could be satisfactorily reproduced assuming a unique iron site, indicating that the four iron ions were equivalent (Fig 1c). Moreover, the nuclear parameters (isomer shift *δ* = 0.42 ± 0.01 mm s^−1^, quadrupole splitting *∆E*_*Q*_ = 1.16 ± 0.05 mm s^−1^, and EFG rhombicity *η* = 0.7 ± 0.1) were similar to those reported for [4Fe-4S]^2+^ clusters with at least three coordinated cysteines [16–18]. Indeed, the shoulder observed at 0.7 mm s^−1^ was better reproduced when considering two different iron sites in a 3:1 ratio (S1 Fig) with nuclear parameters reminiscent of those obtained for the substrate-free aconitase [19]. Interestingly, no [Fe-S] cluster binding was observed on SmsB alone after reconstitution (Fig 1d). In contrast, we could reconstitute an [Fe-S] cluster-bound SmsC (1.7 Fe and 1.6 S/ SmsC monomer (Fig 1d) with spectroscopic properties (UV-visible absorption and Mössbaur spectroscopy) clearly evidencing a [4Fe-4S] diamagnetic cluster (S2 Fig). Altogether, these biochemical and biophysical analyses demonstrated that SmsB and SmsC forms an SmsC_2_B_2_ hetero-tetramer complex that assembles an [4Fe-4S] cluster, probably in the SmsC subunit.

### X-ray structural analysis of the [Fe-S] cluster-free SmsC_2_B_2_ complex

**Fig 2:**
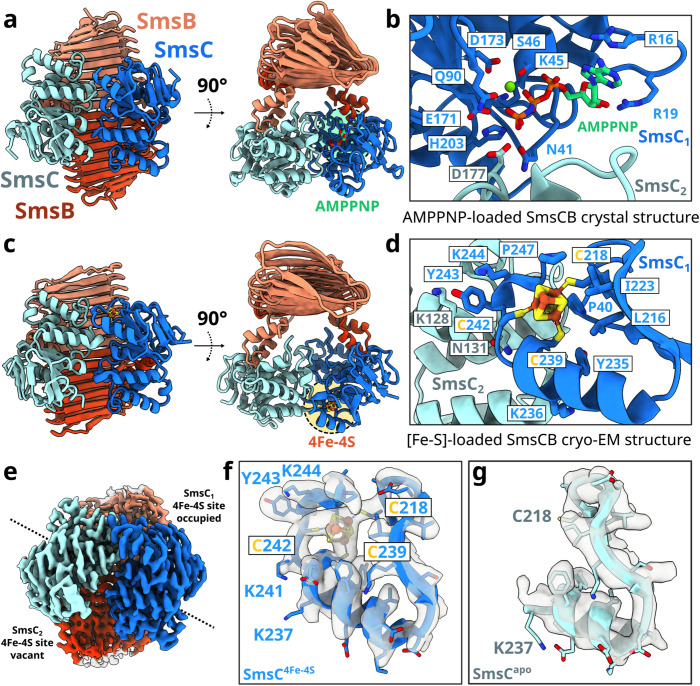
Overall architecture of the SmsC_2_B_2_ complex. **(a)** AMP-PNP-loaded SmsC_2_B_2_ crystal structure. **(b)** Close-up on the binding site of the AMP-PNP/Mg^2+^ showing residues surrounding the binding site of AMP-PNP. **(c)** Cryo-EM structure of the [Fe-S]-bound SmsC_2_B_2_ complex. **(d)** Close-up on the binding site of the [Fe-S] cluster showing the C218, C239, and C242 coordinating the [Fe-S] cluster. The cluster binding site lies in a solvent-exposed hydrophobic pocket consisting of residues P40, L216, I223, Y235, F234, and P247. **(e)** The [Fe-S] cluster-bound SmsC_2_B_2_ complex exhibits asymmetry. **(f)** The [Fe-S] bound SmsC displays a folded C-terminal α-helix composed of residues 227–241 and a short terminal loop. **(g)** Close-up from the apo-SmsC-COOH region till residue 237.

We set up crystallization conditions both under oxic and anoxic conditions and we obtained crystals of SmsC_2_B_2_ in both conditions. To obtain a nucleotide-bound structure, the crystals of SmsC_2_B_2_ grown in oxic conditions were soaked with 10 mM adenosine-5′-[(β,γ)-imido]triphosphate (AMP-PNP). For the [Fe-S] cluster-bound structure, we chemically reconstituted the protein complex, as described above, and performed crystallization inside the anoxic chamber yielding new crystal forms. Unfortunately, colorless crystals and further iron quantitation showed that the crystallized SmsC_2_B_2_ complex had no bound [Fe-S] cluster, but a sulfate anion bound to SmsC. Diffraction data collection and model refinement statistics are summarized in Table 1. Both SO_4_- and AMP-PNP-bound crystal structures of the SmsC_2_B_2_ complex could be superimposed with a r.m.s.d. of 2.5 Å. SmsB and SmsC assembled into a symmetric hetero-tetramer consisting of two subunits of each protein related by a 2-fold symmetry (Fig 2a). The SmsB subunits consist of a right-handed β-helix core domain (residues 33–289) connected by a short linker to a helical hairpin that serves as an anchoring point for SmsC, analogous to the coupling helices of the transmembrane subunits of ABC transporters [20]. The first N-terminal 32 residues of SmsB are disordered and were not included in the final model. However, we observed a weak extra electron density in the different maps, consistent with an *α*-helix likely belonging to this N-terminal region close to the center of the complex (Fig 2a).

**Table 1:** Data collection and refinement statistics.

	SmsC_2_B_2_-AMP-PNP	SmsC_2_B_2_-SO_4_	SmsC_2_-AMP-PN
**Accession code**	**9H7Y**	**9HBL**	**9H7X**
**Wavelength**	1.27819	0.97856	0.95372
**Resolution range**	70.39–3.198 (3.532–3.198)	61.931–2.191 (2.460–2.191)	73.419–2.525 (2.701–2.525)
**Space group**	C 2 2 21	P 1 21 1	P 31
**Unit cell**	85.167 125.038 133.056 90 90 90	75.291 132.733 75.34 90 111.66 90	84.777 84.777 61.232 90 90 120
**Total reflections**	154,202 (6,774)	315,750 (14,200)	124,335 (6,173)
**Unique reflections**	8,240 (412)	42,393 (2,120)	13,066 (653)
**Multiplicity**	18.7 (16.4)	7.4 (6.7)	9.5 (9.5)
**Completeness (%)**	90.6 (57.8)	93.8 (70.7)	91.6 (49.0)
**Mean I/sigma(I)**	7.4 (1.5)	8.9 (1.9)	9.7 (1.2)
**Wilson B-factor**	99.13	52.22	66.49
**R-merge**	0.307 (4.205)	0.113 (0.856)	0.168 (2.151)
**R-meas**	0.317 (4.344)	0.121 (0.928)	0.177 (2.274)
**R-pim**	0.075 (1.075)	0.044 (0.350)	0.057 (0.735)
**CC1/2**	0.992 (0.450)	0.996 (0.770)	0.997 (0.490)
**Reflections used in refinement**	8,229	42,355	13,065
**Reflections used for R-free**	426 (37)	2,101 (7)	614 (58)
**R-work**	0.2681	0.2015	0.1983
**R-free**	0.2844	0.2465	0.2763
**Number of non-hydrogen atoms**	4,210	8,844	3,974
**Macromolecules**	4,165	8,327	3,832
**Ligands**	32	59	55
**Solvent**	13	458	87
**Protein residues**	531	1,062	483
**RMS (bonds)**	0.011	0.012	0.012
**RMS (angles)**	1.39	1.43	1.47
**Ramachandran favored (%)**	94.88	94.78	95.20
**Ramachandran allowed (%)**	4.74	4.84	3.97
**Ramachandran outliers (%)**	0.38	0.38	0.84
**Rotamer outliers (%)**	7.64	4.70	6.46
**Clashscore**	8.33	6.91	8.55
**Average B-factor**	147.30	65.31	81.65
**Macromolecules**	147.50	65.67	82.14
**Ligands**	160.31	67.60	77.28
**solvent**	51.36	58.54	63.16

Statistics for the highest-resolution shell are shown in parentheses.

**Fig 3:**
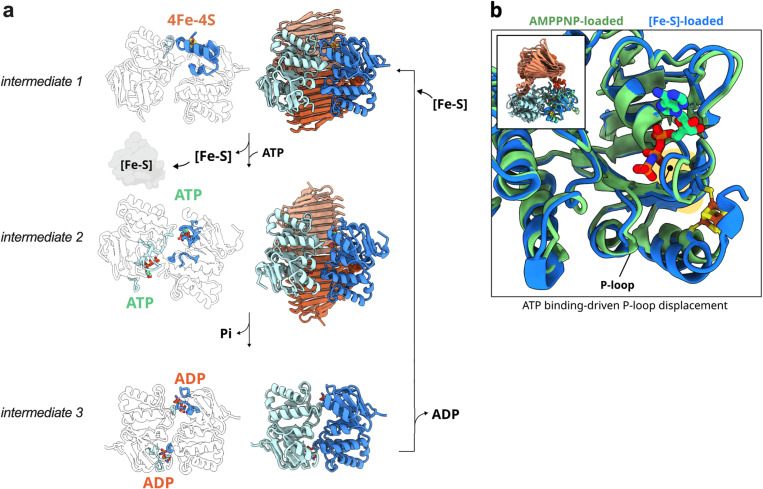
Proposed mechanism of the [Fe-S] biogenesis process on the scaffold SmsC_2_B_2_. SmsC_2_B_2_ assembles first a [Fe-S] cluster (ATP not required), on one of the SmsC subunits (**a**, intermediate 1). Upon interaction, the [Fe-S] cluster is transferred from SmsC_2_B_2_ to the targeted apo-protein client. Upon dissociation of the cluster, the C-terminal extension of SmsC unfolds, destabilizing the asymmetric SmsC-SmsC dimer observed in the cryo-EM structure, and allowing the SmsC_2_B_2_ complex to open to adopt a conformation like the one observed in the crystal structure (**a**, intermediate 2). ATP binding triggers conformational changes in the P-loop (**b**). The SmsC subunits rearrange again to transiently form a tight dimer as seen in the SmsC_2_ structure favoring ATP hydrolysis (**a,** intermediate 3), after which ADP and Pi leave SmsC and SmsC is reset to engage into assembling a new [Fe-S] cluster (a, intermediate 1).

The SmsC subunit, consisting of a RecA-like domain and a helical domain, contains all the structural motifs of the nucleotide-binding (NBD) subunits of the ABC ATPases (i.e., Walker A, Walker B, ABC signature, D-, Q-, and H-loops) [18] (Fig 2b). Most of the interactions between SmsC and the nucleotide are similar to those observed in other NBD structures of ABC transporters [21]. The structure derived from crystals grown in anoxic conditions showed that the nucleotide has been displaced from the ATPase active site by a sulfate anion located into the usual site for the γ-phosphate of ATP. Additionally, SmsC contains a partially folded C-terminal extension comprising a two-cysteine motif (-C-X-X-C-) found in some [Fe-S] cluster-containing proteins of the CIA pathway [22]. The two SmsB subunits tightly interact through an extended dimeric interface, which together with the two SmsB-SmsC contact interfaces stabilizes the hetero-tetrameric assembly [23]. The interface between the SmsC subunits is weaker (the buried surface area is 730 Å^2^), and likely susceptible to changes upon ATP binding and hydrolysis [24]. Such a conformational change is supported by the structure of the SmsC_2_ bound to an AMP-PN, which exhibits a “close” conformational state, similar to that observed in many structures of ABC transporters. This “close” conformation is required for the hydrolysis of ATP as it brings essential catalytic residues of the ABC signature motif close to the nucleotide phosphates [20,21] (see below Fig 3, intermediate 3).

### Cryo-EM analysis identifies the [4Fe-4S] cluster coordination site in one SmsC subunit

Since attempts to crystallize a [Fe-S] cluster-bound protein complex either in the absence or in the presence of AMP-PNP were unsuccessful, we used cryo-EM to determine its structure. The reconstituted SmsC_2_B_2_ complex prepared as described above for crystallization in anoxic conditions, was rapidly vitrified for cryo-EM single particle analysis, and the structure determined reached a global resolution of 2.64 Å. The protein complex prepared in these experimental conditions revealed that the C-terminal residues C218, C239, and C242 of one SmsC subunit coordinate a [4Fe-4S] cluster (Fig 2c). These three cysteine residues bind the cluster in a solvent-exposed hydrophobic pocket consisting of residues P40, L216, I223, Y235, and P247 (Fig 2d). Although the SmsC_2_B_2_ complex could in principle contain two such binding sites at the SmsC head-to-tail dimer interfaces, the two SmsC subunits form a strong asymmetric dimer with a single populated [Fe-S] cluster binding site (Fig 2e). All residues coordinating the cluster and forming the hydrophobic pocket are in a single SmsC chain. In contrast with the unoccupied SmsC subunit (apo-SmsC), the [Fe-S] cluster-bound SmsC displays a folded C-terminal α-helix composed of residues 227–241 and a short terminal loop (Fig 2f and 2g). This region comprises the two-cysteine motif (-C_239_-G_240_-K_241_-C_242_-) coordinating the [Fe-S] cluster, and P247 closing the hydrophobic pocket. Additionally, residues of the C-terminal region extend the dimeric interface between the apo-SmsC and [Fe-S] bound SmsC subunit, in the vicinity of the cluster binding site. This interface consists of a cation–π interaction between the side chains of the K128 in apo-SmsC and the Y243 in [Fe-S] bound SmsC, as well as the side chain-backbone intermolecular hydrogen bonds from the N131 in apo-SmsC and the K236 in [Fe-S] bound SmsC (Fig 2d). As in the crystal structure, the N-terminal of SmsB (residues 1–62) could not be unambiguously resolved in the cryo-EM map of the SmsC_2_B_2_ complex.

**Fig 4:**
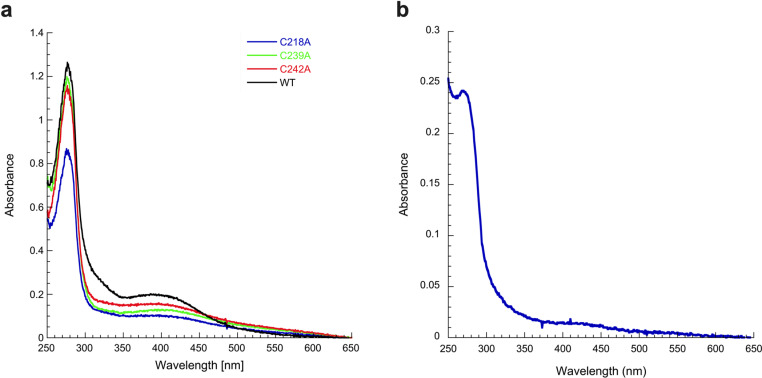
Residues C219, C239, and C242 of SmsC act as ligands of the [Fe-S] cluster. **(a)** SmsC (84 μM) (black), SmsC_C239A_ (84 μM) (green), SmsC_C242A_ (84 μM) (red), and SmsC_C218A_ (56 μM) (blue) were incubated with 5 equivalents of Fe^2+^/SmsC, 5 equivalents of Na_2_S/SmsC, and 3 mM DTT. UV-Vis absorption spectrum of SmsC shows an absorption at 420 nm compared to the variants SmsC_C218A_, SmsC_C239A_, and SmsC_C242A_. **(b)** SmsC_(C239A-C242A)2_B_2_ (50 μM) was incubated with 5 equivalents of Fe^2+^/SmsC_(C239A-C242A)2_B_2_, 5 equivalents of Na_2_S/SmsC_(C239A-C242A)2_B_2_, and 2 mM DTT. UV-Vis absorption spectrum of the SmsC_(C239A-C242A)2_B_2_variant shows no absorbance at 420 nm (1 nmol of SmsC_(C239A-C242A)2_B_2_ contains 0.23 nmol of iron and 0.3 nmol of sulfur). The data underlying this figure can be found in Fig 4 and S2 Data.

The C218, C239, and C242 residues of SmsC were tested for their role in coordinating the [4Fe-4S] cluster. The SmsC_C218A_, SmsC_C239A_, SmsC_C242A_, and SmsC_C239-C242A_B variants were constructed, purified, and submitted to chemical reconstitution assay (see Table 2). UV-visible spectroscopy of all single-cysteine variants revealed a decreased absorbance at 420 nm, consistent with impaired [Fe-S] cluster coordination (Fig 4a). Despite this reduction, these variants retained high level of iron and sulfur content (Table 2). The double variant SmsC_(C239-C242A)2_B_2_ also exhibited a much-diminished absorbance at 420 nm (Fig 4b) while quantitative analysis of metal content indicated that this variant retained only 0.23 ± 0.2 nmol of Fe and 0.3 ± 0.1 nmol of S per nmol of protein complex (Table 2). Together, these results demonstrated that C218, C239, and C242 residues are critical ligands for coordinating a [4Fe-4S] cluster under the tested conditions.

**Table 2:** Iron/sulfide content of each reconstituted protein/protein complex.

	Fe content (nmol)	S content (nmol)
**SmsC**_**2**_ **B**_**2**_	2.5	2.4
**SmsC**	1.7	1.6
**SmsC**_**K45R**_	1.4	1.6
**SmsC**_**C218A**_	2.2	2.4
**SmsC**_**C239A**_	2.4	2.6
**SmsC**_**C242A**_	3	2.2
**SmsC**_**(C239A/C242A)2**_ **B**_**2**_	0.23	0.3
**AMP-PCP-SmsC**_**2**_ **B**_**2**_	0.21	0.29

### SmsC is an ATPase

**Fig 5:**
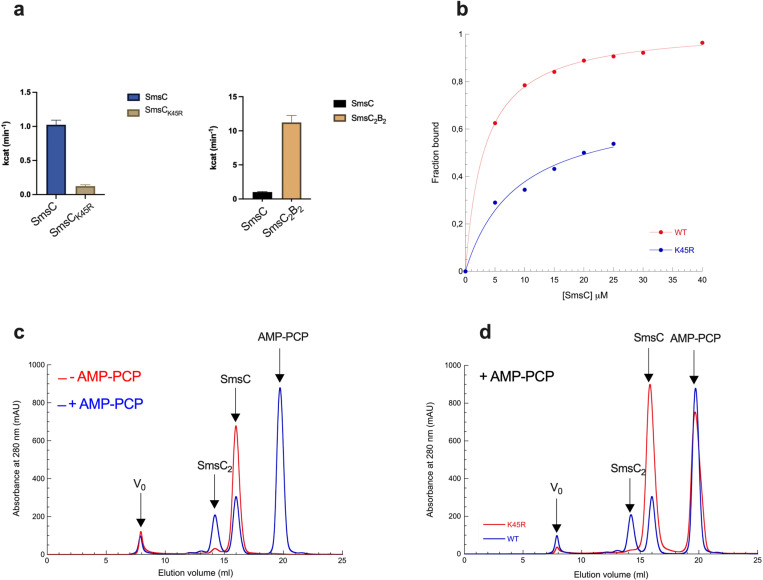
Role of ATP binding/hydrolysis in SmsCB. **(a)** SmsC (35 µM), SmsC_K45R_ (35 µM), and SmsC_2_B_2_ (35 µM) proteins were added to 1 ml of 25 mM Hepes buffer (pH 7.6) containing 100 mM KCl, 5 mM MgSO_4_, 5 mM phospho-enol pyruvate, 1 mM NADH, 5 UI of PK, and 10 UI of LDH. Then, 1 mM of ATP was added to initiate the reaction at 25 °C. Specific activities were calculated using the molar extinction coefficient of 6.22 mM^−1^ cm^−1^ for NADH and the protein concentrations determined from the extinction coefficient. SmsC_2_B_2_ displays an ATPase activity 10-fold higher than SmsC. SmsC_K45R_ displays a residual ATPase activity. **(b)** Equilibirum binding curve. SmsC proteins from 0 to 40 µM and SmsC_K45R_ proteins from 0 to 25 µM were incubated with 400 nM concentration of mantATP*y*S in HEPES buffer 25 mM, KCl 100 mM, MgSO_4_ 5 mM, pH 7.6. Measurements were taken using a fluorimeter (*λ*_exc_ 355 nm and *λ*_em_ 448 nm for mantATP*y*S). Dissociation constant was calculated using the equation *y* = *m*1 * *x*/(*m*2 + *x*). **(c)** SmsC proteins first incubated with 10 mM AMP-PCP (blue) or not (red) were purified by SEC. Elution peaks of SmsC, SmsC_2_, AMP-PCP Sms are indicated on top of the graph. **(d)** SmsC (blue) and SmsC_K45R_ (red) proteins were incubated with 10 mM of AMP-PCP and purified by SEC. Elution peaks of SmsC, SmsC_2_, AMP-PCP Sms are indicated on top of the graph. The data underlying this figure can be found in Fig 5 and S3 Data.

SmsC harbors Walker A/B signatures of ABC ATPases. To obtain further functional insights, we performed a thorough enzymological analysis of the influence of ATP on SMS. Whereas SmsC exhibited ATPase activity with a k_cat_ of 0.98 min^−1^ a variant impaired in the ATP binding site (Walker A motif), SmsC_K45R_, displayed a weak ATPase activity with a k_cat_ of 0.13 min^−1^ (Fig 5a). The complex SmsC_2_B_2_ displayed an ATPase activity approximately 10-fold higher than SmsC alone (10 min^−1^), indicating that SmsB stimulated the ATPase activity of SmsC (Fig 5a). Moreover, ligand binding experiments using mantATP*y*S showed a Kd approximately three times higher for the variant SmsC_K45R_ (8.76 ± 2 nM) than for the wild-type SmsC (3.2 ± 0.2 nM) (Fig 5b). Incubation of SmsC with excess of adenosine-5′-[(β,γ)-methyleno]triphosphate (AMP-PCP) led to SmsC dimer formation (Fig 5c). The SmsC_K45R_ variant was not able to form homodimers upon AMP-PCP binding (Fig 5d), consistent with the notion that binding of ATP is required for dimerization of SmsC.

### ATP and [Fe-S] cluster binding are mutually exclusive

**Fig 6:**
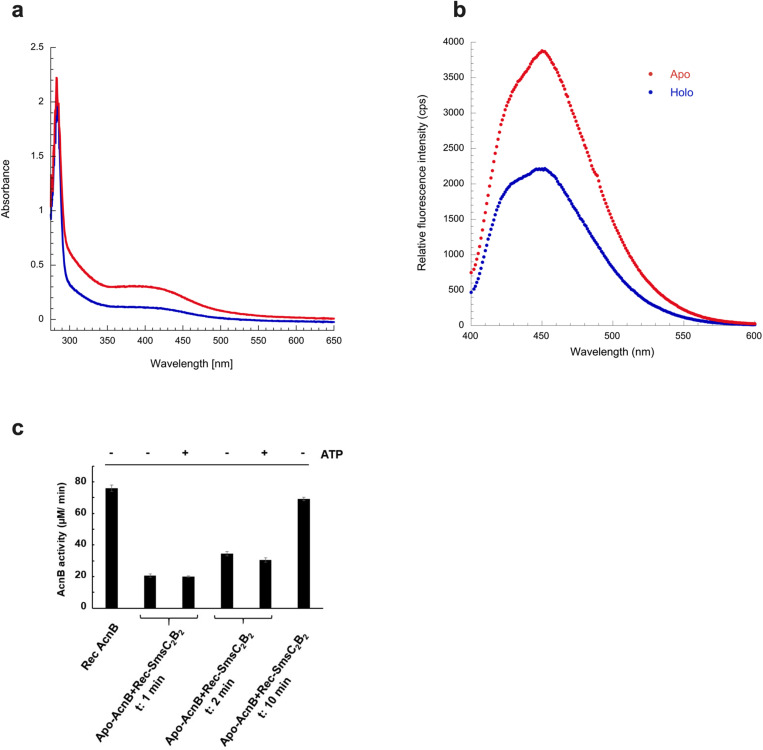
ATP and [Fe-S] cluster binding are mutually exclusive. **(a)** SmsC_2_B_2_ (84 μM) (red) and AMP-PCP-SmsC_2_B_2_ (84 μM) (blue) were incubated with 5 equivalents of Fe^2+^/ SmsC_2_B_2_, 5 equivalents of Na_2_S/ SmsC_2_B_2_, and 3 mM DTT. UV-Vis absorption spectrum of SmsC_2_B_2_ shows an absorption at 420 nm compared to the AMP-PCP-SmsC_2_B_2_ bound form. **(b)** Relative fluorescence measurements. SmsC_2_B_2_ (50 μM) (red) and [Fe-S]-SmsC_2_B_2_ (50 μM) (blue) were incubated with 21 μM of mantATP*y*S. Measurements were taken using a fluorimeter (*λ*_exc_ 355 nm and *λ*_em_ 448 nm). **(c)** Apo-AcnB (0.5 nmol) was incubated with reconstituted SmsC_2_B_2_ (1.74 eq.; 2.9 Fe/3.0 S per SmsC_2_B_2_) ± 1 mM ATP and 2 mM MgCl₂. Aconitase activity was measured after 1, 2, and 10 min to assess the effect of ATP on [Fe-S] cluster transfer. As a positive control (RecACnB), apo-AcnB was assayed after 30 min with 5 molar excess of Fe^2+^ and S^2−^ in the presence of 500 µM DTT. The initial velocity (μM isocitrate/min) was measured in duplicate, short bars correspond to mean deviation. The data underlying this figure can be found in Fig 6 and S4 Data.

Next, we explored whether [Fe-S] cluster assembly and ATP binding were connected. For this, we asked if we could reconstitute a [Fe-S] cluster onto AMP-PCP-SmsC_2_B_2_ bound protein. All our attempts failed, suggesting that the presence of AMP-PCP prevented the binding of the [Fe-S] cluster (Fig 6a). Subsequent quantitation revealed that iron and sulfur content per mol of complex are 0.21 ± 0.3 Fe and 0.29 ± 0.12 S per AMP-PCP-SmsC_2_B_2_ complex. Conversely, reconstituted [Fe-S]-SmsC_2_B_2_ was incubated with mantATP*y*S. No increase in fluorescence intensity was observed, indicating that mantATP*y*S could not bind [Fe-S]-SmsC_2_B_2_ (Fig 6b). Last, UV-visible spectroscopy data showed that adding ATP failed to alter [Fe-S]-bound SmsC_2_B_2_ stability under oxic conditions, probably because ATP could not bind on [Fe-S]-SmsC_2_B_2_. Altogether these results showed that binding of ATP and [Fe-S] clusters are mutually exclusive. Interestingly, no difference in the capacity of SmsC_2_B_2_ to transfer a [4Fe-4S] cluster on apo-aconitase in vitro was observed whether ATP was present or not (Fig 6c). The SmsC_K45R_ variant altered in the Walker A box showed impaired ATPase activity, yet retained the capacity to bind a [4Fe-4S] cluster (S3 Fig). Altogether these data revealed an unexpected connection between ATP binding and [Fe-S] cluster coordination, pointing to a coupling in the interaction of SmsC with both of its substrates.

### A mineral sulfur source mediates cluster formation by SmsCB in vivo

**Fig 7:**
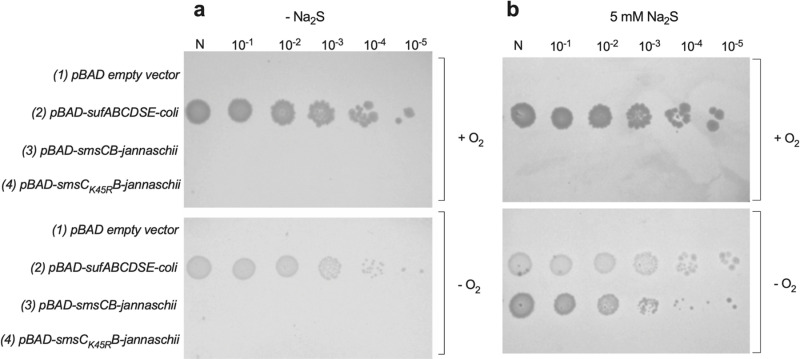
Sodium sulfide is the source of sulfur for the SmsCB complex. Spot test assay for growth indifferent culture dilutions of *E. coli. ΔiscUAΔsuf* MEV carrying the empty pBAD vector (lane 1), the pBAD vector carrying the *Escherichia coli sufABCSDE* operon (lane 2), the pBAD vector carrying the *Methanocaldococcus jannaschii smsCB operon* (lane 3), and the pBAD vector carrying the *M. jannaschii smsC*_*K45R*_*B operon* (lane 4). Medium was LB supplemented with 0.2% arabinose **(a)** or 0.2% arabinose and 5 mM Na_2_S **(b)**, in oxic or anoxic conditions.

No cysteine desulfurase encoding genes are present in the SMS encoding operon or anywhere else in the genome of *M. jannaschii*, raising the question of the source of sulfur for feeding the SMS system. Early work has proposed that H_2_S could act as a sulfur source for [Fe-S] proteins in *Methanococcus maripaludis* [25]. To investigate whether H_2_S could be a *bona fide* sulfur source for *M. jannaschii* SMS system as well in vivo, we tested the capacity of the *M. jannaschii smsCB* operon to complement an *E. coli* FBE605 (Table 3) recipient strain lacking both functional ISC and SUF machineries. FBE605 strain has *∆iscUA∆sufABCDSE* deletions, which renders it non-viable as it cannot maturate the isoprenoid synthesizing [Fe-S] containing IspG and IspH proteins. Previously, FBE605 strain was made viable by bringing an ectopically version of eucaryotic genes encoding an arabinose-controlled, mevalonate (MVA)-dependent [Fe-S] independent isoprenoid synthesizing pathway [26,27]. Thus, the resulting FBE605 strain can grow if both arabinose and MVA are present in the medium or if a [Fe-S] producing system is provided. We observed that the *M. jannaschii smsCB* operon was indeed able to rescue viability of the *∆iscUA∆sufABCDSE* strain, but only if Na_2_S was added to the medium and the strains incubated under anoxic conditions (Fig 7a and 7b). Importantly, the *M. jannaschii smsC*_*K45R*_*smsB* operon, defective in ATPase activity, was unable to complement the *E. coli ∆iscUA∆sufABCDSE* strain. It is worth mentioning that substituting Na_2_S for l-cysteine did not enable complementation, either under aerobic or anaerobic conditions (S4 Fig). Altogether, these results demonstrated that SMS acts as a [Fe-S] biogenesis system in vivo and strongly support the view that it uses sulfide as a source of sulfur.

**Table 3:** *E. coli* strains, plasmids, and oligonucleotides used in this study.

Strain or plasmid	Relevant genotype or characteristics	Reference or source
***E. coli* strains**		
DH5α	Cloning strain	Lab collection
FBE605 *∆iscUA∆suf* MEV	*MG1655 ΔlacZ PiscR (trans)::lacZ Δsuf MEV::kn ΔiscAU*	Lab collection
FBE682	MG1655 F^−^(λDE3) *ilvG rfb50 rph1*	M. Cashel gift
**Plasmids**		
**pEB1188**	**pET-6his-Tev** Amp^R^, T7 promoter, N-terminal 6His-Tev	[39,40]
**pVP334**	**pET-6his-Tev-*smsC-jannaschii*** Obtained by PCR with ebp433/ebp434 on synthetic fragment cloned in pEB1188 (EcoRI/XhoI)	This study
**pVP382**	**pET-6his-Tev-*smsB-jannaschii*** Obtained by PCR with ebp506/ebp388 on synthetic fragment cloned in pEB1188 (SacI/XhoI)	This study
**pVP299**	**pET-6his-Tev-*smsCB-jannaschii*** Obtained by PCR with ebp416/ebp388 cloned on synthetic fragment cloned in pEB1188 (SacI/XhoI)	This study
**pVP373**	**pET-6his-Tev-*smsC***_***K45R-***_***jannaschii*** pVP334 derivative containing a *smsC*_*K45R*_ *allele*	This study
**pVP367**	**pET-6his-Tev-*smsC***_***C239A/C242A***_***B-jannaschii*** pVP299 derivative containing a *smsC*_*C239A/C242A*_*B* operon	This study
**pVP428**	**pET-6his-Tev-*smsC***_***C239A-***_***jannaschii*** pVP334 derivative containing a *smsC*_*C239A*_ *allele*	This study
**pVP430**	**pET-6his-Tev-*smsC***_***C242A-***_***jannaschii*** pVP334 derivative containing a *smsC*_*C242A*_ *allele*	This study
**pVP487**	**pET-6his-Tev-*smsC***_***C218A-***_***jannaschii*** pVP334 derivative containing a *smsC*_*C218A*_ *allele*	This study
**pGSO164**	whole suf operon under Bad promoter, Amp^R^	Lab collection
**pBAD24**	Amp^R^, pBR322 promoter	[41]
**pVP274**	**pBAD-*smsCB-jannaschii*** Obtained by PCR with ebp388a/ebp388b on synthetic fragment cloned in pBAD24 (NcoI/SalI)	This study
**pVP498**	**pBAD-*smsC***_***K45R***_***B-jannaschii*** pVP274 derivative containing a *smsC*_*K45R*_*B* operon	This study
**Oligos name**	**Sequence (5′–3′)**	**Reference**
FW *smsC M. jannaschii* SacI (Ebp416)	TTCGAGCTCATGGTCTCTATTATGTTACTTAAGGTAG	This study
FW *smsC M. jannaschii* EcoRI (Ebp433)	ACTGAATTCATGGTCTCTATTATGTTACTTAAGGTAG	This study
RV *smsC M. jannaschii* XhoI (Ebp434)	ACGCTCGAGTTACTTTCCGTCTGGAACCTTCTTG	This study
FW *smsB M. jannaschii* SacI (Ebp506)	TCCGAGCTCATGTCGATCAAGGAAGAGCTGATG	This study
RV *smsB M. jannaschii* XhoI (Ebp388)	ACGCTCGAGTTACAGATCGCCGATCATACCC	This study
FW *smsC M. jannaschii* NcoI (Ebp388a)	TTCACCATGGTCTCTATTATGTTACTTAAGGTAG	This study
RV *smsB M. jannaschii* XhoI (Ebp388b)	ACGCTCGAGTTACAGATCGCCGATCATACCC	This study
FW *smsC*_*C239A/C242*_ *M. jannaschii* (Ebp490)	GGAGAGTTCTACAAGAAGGAGGCTGGTAAGGCTTACAAGAAGGTTCCAGACGG	This study
RV *smsC*_*C239A/C242*_ *M. jannaschii* (Ebp491)	CCGTCTGGAACCTTCTTGTAAGCCTTACCAGCCTCCTTCTTGTAGAACTCTCC	This study
FW *smsC*_*K45R*_ *M. jannaschii* (Ebp502)	GTCCTAATGGTGCCGGTAGGTCGACTTTAGCCTAC	This study
RV *smsC*_*K45R*_ *M. jannaschii* (Ebp503)	GTAGGCTAAAGTCGACCTACCGGCACCATTAGGAC	This study
FW *smsC*_*C239A*_ *M. jannaschii* (OMD5)	GAGAGTTCTACAAGAAGGAGGCTGGTAAGTGTTACAAGAAGG	This study
RV *smsC*_*C239A*_ *M. jannaschii* (OMD6)	CCTTCTTGTAACACTTACCAGCCTCCTTCTTGTAGAACTCTC	This study
FW _*smsCC242A*_ *M. jannaschii* (OMD7)	CAAGAAGGAGTGTGGTAAGGCTTACAAGAAGGTTCCAGAC	This study
RV *smsC*_*C242A*_ *M. jannaschii* (OMD8)	GTCTGGAACCTTCTTGTAAGCCTTACCACACTCCTTCTTG	This study
FW *smsC*_*C218A*_ *M. jannaschii* (OMD13)	CAGACCGTGTATCATTGATCGCTGCCGGTGAGGTCAT	This study
RV *smsC*_*C218A*_ *M. jannaschii* (OMD14)	ATGACCTCACCGGCAGCGATCAATGATACACGGTCTG	This study
T7	TAATACGACTCACTATAGGG	This study
T7term	CTAGTTATTGCTCAGCGGT	This study
pBAD-FP	ATGCCATAGCATTTTTATCC	This study
pTrcHis-RP	CTGATTTAATCTGTATCAGG	This study

Restriction sites are underlined.

### Diversity and [Fe-S] cluster binding capacities of SmsC C-terminal region

**Fig 8:**
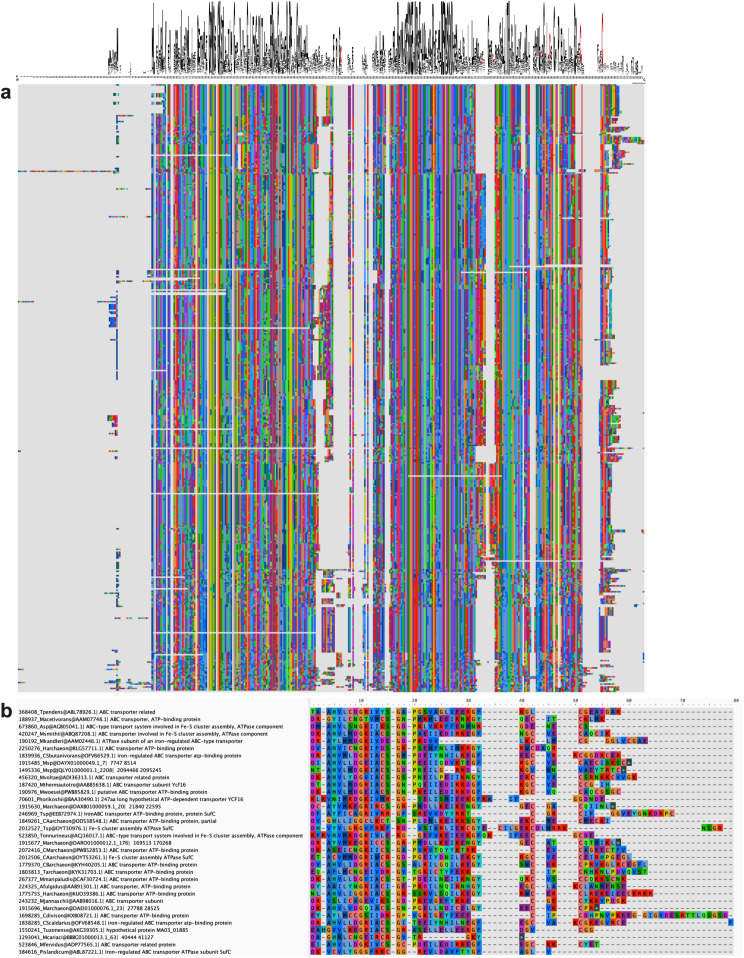
Diversity of the C-terminal region (CTR) of SmsC. **(a)** Multiple sequence alignment of SmsC sequences showing that the beta-beta-alpha region is well conserved from D212 to G240 residues. Number of cysteine residues per site in the multiple alignement of 1,387 SmsC. **(b)** Subsample of SmsC CTR showing the diversity in terms of cysteine arrangements.

**Fig 9:**
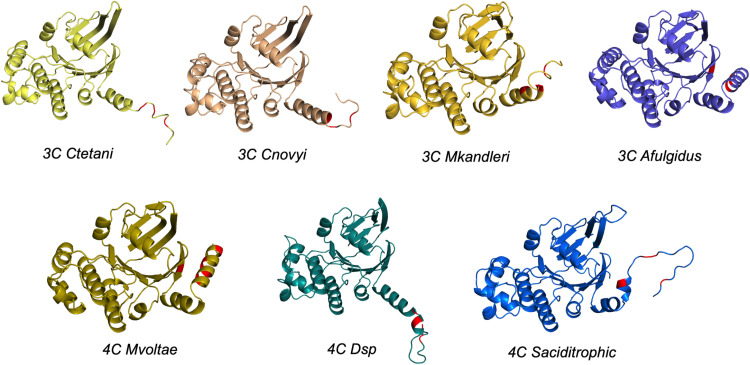
Alphafold predictions of the structures of the Sms CTRs harboring different arrangement of Cys residues. Predictions harbor several Cys residues close to each other in the structural models, being compatible with a [Fe-S] binding cluster capacity. Alphafold prediction of the SmsCB proteins of *Clostridium tetani (Ctetani)*, *Clostridium novyi (Cnovyi)*, *Methanopyrus kandleri (Mkandleri)*, *Archaeoglobus fulgidus (Afulgidus), Methanococcus voltae (Mvoltae), Dehalobacter sp (Dsp)*, and *Syntrophus aciditrophicus (Saciditrophic)***.** Cysteine residues are shown in red.

Based upon the structural analysis of SmsC described above, we defined a C-terminal region (referred to as CTR below) ranging from residue D212 to the COOH terminus, including the beta-beta-alpha region. A multiple sequence alignment of archaeal SmsC sequences showed that the beta-beta-alpha region is relatively conserved from D212 to G240 residues (Fig 8a) whereas the extreme C-terminal region is highly variable (Fig 8a). About 75% of archaeal SmsC sequences (292 out of 415) were found to harbor the C-X-X-C motif (see above). Interestingly, Cys residues were found to be enriched in the CTR (Fig 8b) but vary in number (from 0 to 5), position, and spacing. Alphafold predictions of the structures of the Sms CTRs harboring different arrangements of Cys residues showed they all harbor several Cys residues close to each other in the structural models, being compatible with a [Fe-S] binding cluster capacity (Fig 9). This analysis revealed that, despite a large variability in sequence, the capacity to bind a [Fe-S] cluster is likely to be a conserved feature of SmsC proteins.

## Conclusions

Previously we identified two minimal [Fe-S] biogenesis systems, MIS and SMS, which we proposed to be ancestors of modern ISC, NIF, and SUF. The MIS system had already received some attention, although under a different appellation [28,29]. In the present study, we focused on SMS from the archaeon *M. jannaschi*. SMS is a two-component system, proposed to be the ancestor of SUF, which comprises six components. SufSE acts as heterodimeric cysteine desulfurase. SufC, an ABC ATPase, which, with SufB and D paralogs, forms a heteromeric scaffold assembling [Fe-S] cluster. SufA, acts as a carrier delivering [Fe-S] clusters to cellular client targets. *M. jannaschi* SmsC and SmsB share 32% and 14% sequence identity with *E. coli* SufC and SufB, respectively, and SmsC, like SufC, harbors Walker A/B signatures of ABC ATPases. Thus, the question is to know how in the absence of a cysteine desulfurase (SufSE), of one of the scaffold component (SufD) and of a carrier (SufA), does SmsCB make and distribute [Fe-S] clusters.

Globally, the three-dimensional structure of the SmsC_2_B_2_ complex from *M. jannaschii* is similar to that of the SufBC_2_D complex from *E. coli* [30]. However, a major and unexpected finding based upon our structural, spectroscopic, and mutagenesis analyses is the demonstration that the [Fe-S] cluster binding site on SmsC_2_B_2_ complex locates in SmsC and in addition in only one SmsC subunit. This departs from the SufBC_2_D system wherein the [Fe-S] cluster is thought to bind at the SufBD interface [30,31], although no conclusive structural study has been produced to date supporting this claim. Despite several attempts, we obtained no evidence for [Fe-S] binding onto SmsB. Moreover, preventing SmsC to bind [Fe-S] cluster by mutagenesis was sufficient to hamper [Fe-S] binding by the whole SmsC_2_B_2_ complex. We found that the three Cys residues, C218, C239, and C242 coordinate the [4Fe-4S] cluster. Given the solvent-exposed location of the assembly site, a solvent molecule might contribute to additional liganding. The C239 and C242 residues are part of the C-terminal unstructured and flexible CTR, which is enriched in C-X-X-C motifs, in all SmsC sequences examined. Interestingly, CTR sequences show a wide diversity, yet we predict that CTR are very likely to enable cognate SmsC to bind [Fe-S] clusters. This is another striking difference from SufC, which has no such flexible CTR. Another specificity of SmsC_2_B_2_ lies in the fact that it assembles a single [4Fe-4S] cluster per complex. This departs from SufBC_2_D, NifU, or IscU, which assemble a [2Fe-2S] or a [3Fe-3S] cluster [30–35]. The reason why orthodox scaffolds bind [2Fe-2S] clusters lies in their capacity to cooperate with A-type carriers [33]. These carriers receive two [2Fe-2S] clusters from the scaffold and convert them to [4Fe-4S] clusters which they eventually transfer to apo-targets. *E. coli* A-type carriers include IscA, SufA, ErpA, and NfuA proteins. Analysis of the genome of *M. jannaschii* failed to identify such A-type carriers. Hence, it is possible that SmsC_2_B_2_ directly transfers the [4Fe-4S] cluster to its target, independently of a carrier. Alternatively, SmsC_2_B_2_ might team up with Mrp, a putative scaffold/carrier whose role remains ill-defined and for which a cognate structural gene was identified in *M. jannaschii* genome [14]. Another issue concerns the maturation of [2Fe-2S] clusters proteins if such species arise at all in *M. jannaschii*. This is a fully open question as only [4Fe-4S] proteins have been characterized in this organism or other related archaea and it is impossible to predict from genome analysis whether a given protein harbors a [2Fe-2S] or [4Fe-4S] cluster.

Our biochemical and structural data indicated that [Fe-S] cluster binding to one SmsC subunit induced a strong asymmetry within its dimeric interface by folding the C-terminal helical region. Thus, we propose that SmsC_2_B_2_ assembles first a [Fe-S] cluster, on one of the SmsC subunits (Fig 3a, intermediate 1). Upon interaction the [Fe-S] cluster is transferred from SmsC_2_B_2_ to the targeted apo-protein client. Upon dissociation of the cluster, the C-terminal extension of SmsC unfolds, destabilizing the asymmetric SmsC-SmsC dimer observed in the cryo-EM structure, and allowing the SmsC_2_B_2_ complex to open up to adopt a conformation like the one observed in the crystal structure (Fig 3a, intermediate 2). ATP binding to this conformer triggers conformational changes in the P-loop (Fig 3b). The SmsC subunits rearrange again to transiently form a tight dimer as seen in the SmsC_2_ structure favoring ATP hydrolysis (Fig 3a, intermediate 3), after which ADP and Pi leave SmsC and SmsC is reset to engage into assembling a new [Fe-S] cluster (Fig 3a, intermediate 1).

The present study uncovered unexpected functional coupling between the binding of ATP and the assembly of [Fe-S] cluster in SmsC. AMP-PCP-bound SmsC_2_B_2_ was unable to accommodate the [Fe-S] cluster and mantATP*y*S could not bind [Fe-S]-SmsC_2_B_2_. This finding provided evidence that the binding of ATP and the [Fe-S] cluster to SmsC_2_B_2_ are mutually exclusive. Interestingly, mutual exclusion between [Fe-S] and the ATP binding sites might be due to their close vicinity in the structure. The physiological value of alternated bindings of ATP and Fe-S clusters might be to serve as a homeostatic control. [Fe-S] proteins being key in energetic producing pathways, one might surmise that when enough [Fe-S] proteins are functioning, ATP level is high and SMS activity ought to be reduced. On the contrary, a reduced level of ATP, reflecting reduced activities of [Fe-S] proteins, will cease to antagonize [Fe-S] assembly on SmsCB, enhanced [Fe-S] biogenesis will ensue and ATP level will be replenished.

A major difference of SMS with respect to the four well-studied [Fe-S] cluster biogenesis machineries, including the other ancestor minimal MIS system, is that there is no identified cysteine desulfurase component in *M. jannaschii* genome. This raises the question of the source of sulfur. Here, we showed that adding Na_2_S in culture media allows SMS to carry out [Fe-S] biogenesis in an *E. coli* strain lacking its own biogenesis systems, ISC and SUF, indicating that SMS can use sulfide for building [Fe-S] clusters. How *E. coli* integrates exogenously provided sulfide into its sulfur metabolism and in particular how sulfide is taken up remain unclear. Previous work by the Imlay lab [36] argued that sulfide freely equilibrates across membranes. Also, a recent transciptomic analysis [37] highlighted the role of sulfide sensing *ygaV* transcriptional regulator in *E. coli* grown under oxygen-limiting and/or sulfide-enriched conditions. Expression of a large number of genes was found modified in the presence of sulfide. Thus, an exciting possibility is that some of these genes might contribute to the SmsCB-mediated complementation reported here and this is being investigated in our lab. This result is consistent with the early proposal that SMS-containing archaea growing in H_2_S-rich environments might build [Fe-S] clusters using exogenous sulfide as a sulfur source [25,38]. Interestingly, we observed complementation only under anaerobiosis, whereas the SUF system is able to mature proteins under both aerobiosis and anaerobiosis. This further supports the hypothesis that SMS operated in anaerobic prokaryotes and that SUF evolved from it as adaptation to an increasingly oxygenic atmosphere after the GOE.

## Materials and methods

### Bacterial strains and plasmid construction

The *E. coli* strains, plasmids, and oligonucleotides used in this study are listed in Table 3. Bacterial strains were routinely grown in aeration at 37 °C in Luria–Bertani broth (LB). When required, ampicillin was added at 100 μg ml^−1^. The nucleotide sequences were codon-optimized for expression in *E. coli* and genes have been ordered from Twist Bioscience (TWB) (Table 4).

**Table 4:** List of SmsB and SmsC protein sequences used for functional analysis.

Organism	SmsB sequence	SmsC sequence
*Methanocaldococcus jannaschii*	MSIKEELMEIIEAIKYTSEKPEEIVHGKGPRIIVKESRIIDVQGDEGIILEGKEEDGKIKAKIIVKKGYKFKYPIHMCFGITEENISQIIDVEIILEEDSSISLMSHCSFPKGKGIKHIMNGIIKIGKNAKFSYNEFHYHGMDGDILVKPTVKVEIDEGGIYISNFTLTKGRIGTLDIEQEIIAKKDAIIDITTRTYAIKEDVVKVNEVVKLNGENAKCIIKSRGAAMDNSKISLKLKIEGNAPYSKGHIDCAEIVKGNAEVESIPIVVVRDDKARITHEAAIGSVDKKQLETLMAKGLDEDEATEIIVKGMIGDL	MVSIMLLKVEDLHVYRGNREILKGVNLTVEENEIHAIIGPNGAGKSTLAYTIMGISGYKPTKGRIIFKGVDIIDKNITERARMGMTLAWQEPARFEGIKVKNYLMLGMNEKYKKDKEIAEEKIREALKLVNLDPDKYLDRYVDETLSGGERKRIELASIICMEPDLAILDEPDSGIDIVSFDEIKRVFDYLKDKGCSLLVITHREELAEHADRVSLICAGEVIKSGDPKEVGEFYKKECGKCYKKVPDGK

For purification experiments, the *smsC* and *smsB* genes of *M. jannaschii* were cloned in the pET6His-Tev vector [39,40]. These constructions enabled the production of N-terminal 6His-tagged SmsC and N-terminal 6His-tagged SmsB proteins, respectively. For complementation experiments, the *smsCB* genes of *M. jannaschii* were cloned in the pBAD [41]. Finally, all the plasmids were sequenced using Eurofins oligonucleotides T7 and T7term for pET vectors or pBAD-FP and pTrcHis-RP oligonucleotides for pBAD vectors.

### Site-directed mutagenesis by PCR

Site-directed mutagenesis was performed by PCR with the Stratagene *pfuUltra* enzyme, the plasmid to be mutated as atemplate, and two complementary oligonucleotides containing the mutation. The amplification products were digested with 10 units of DpnI enzyme before being used for transformation into NEB5α.

### In vivo complementation assay

The strain MG1655 *∆iscUA∆sufABCDSE* (FBE605) is a derivative of *E. coli* MG1655 in which *iscUA* and *sufABCDSE* genes containing regions are deleted and which contains eucaryotic genes encoding MVA-dependent isoprenoid synthesis pathway [26,27,42]. This strain is viable on LB supplemented with 0.2% arabinose and 0.5 mM MVA (mevalonolactone, Sigma-Aldrich). FBE605 was sequentially transformed with pBAD plasmids derivatives (Table 3). Transformants were selected aerobically at 37 °C on LB agar in the presence of MVA and arabinose. Cells were pelleted, washed, resuspended in LB, and used as a normalization of OD_600_ = 1. Serial dilutions in 1 ml final volume were performed until 10^−6^, and 2 μl drops were plated on LB plates supplemented or not with 5 mM Na_2_S. For anaerobic growth, plates were placed in a jar with AnaeroGen bags from ThermoScientific.

### Expression and purification of SmsB and SmsC from *M. jannaschii*

SmsB and SmsC proteins were produced in strain MG1655(DE3) (FBE682) growing under oxic conditions in LB medium. Expression was carried out in MG1655(DE3) at 30 °C until OD_600_ reached 0.8–0.9, and induced at 28 °C with 0.1 mM IPTG during 3 h. Pellets were stored at −20 °C. Harvested cells were resuspended in washing buffer (25 mM Tris-HCl pH 7.8, 100 mM NaCl, 25 mM imidazole). Cells were lysed by Cell Disruption System at 25,000 Psi and the lysate was recovered with MgCl_2_ 5 mM and benzonase at 6,240 UI. Then, the lysate was cleared by centrifugation (12.000*g*, 20 min, 4 °C). Supernatants were loaded onto a 5 ml HisTrap HP (Cytiva). The column was washed using buffer (25 mM Tris-HCl pH 7.8, 100 mM NaCl, 25 mM imidazole) and proteins were eluted with washing buffer containing 300 mM imidazole. Fractions containing SmsC and/or SmsB proteins were pooled and dialyzed overnight in 50 mM Tris-HCl pH 7.8, 100 mM NaCl, 1 mM dithiothreitol (DTT) buffer. Treatment with recombinant TEV protease was also added to remove the N-terminal His-tag. The dialysate was recovered and loaded onto a His-binding (Ni-NTA-Agarose) pre-equilibrated with 50 mM Tris-HCl pH 7.8 and NaCl 10 mM. The eluate was concentrated using PES column 10,000 MWCO. This was injected on a calibrated Hiload 600 Superdex 200 column (50 mM Tris-HCl pH 7.8, 100 mM NaCl, 1 mM DTT). SmsB was mainly homodimeric in solution (71,200 kDa) and SmsC monomeric (28,600 kDa). Then, SmsB_2_ et SmsC proteins were mixed with molar amounts corresponding to SmsC_2_B_2_ and incubated overnight, before injecting again on a Hiload 600 Superdex 200 column (50 mM Tris-HCl pH 7.8, 100 mM NaCl, 1 mM DTT). From this size exclusion chromatography, resulted different peaks, corresponding to the reformed SmsC_2_B_2_ complex (128,400 Da) and SmsC (28,600 Da) that could be in excess or have been detached from the complex itself. Desired fractions were combined, concentrated, aliquoted, and checked for purity by SDS–PAGE. Each time, analysis of the size exclusion chromatography fractions by SDS-PAGE revealed the protein purity. Protein concentration was determined via UV-visible spectroscopic analysis with Jasco V-730 spectrophotometer, by using *ε*_280_ = 0.714 M^−1^ cm^−1^ and *ε*_280_ = 0.335 M^−1^ cm^−1^ for SmsC and SmsB, respectively. The purification yield was approximately 13 mg/L for SmsC_2_ and 8 mg/mL for SmsB. The UV-visible absorbance spectrum of protein purified under oxic conditions presented no characteristic absorption bands indicative of a metal-bound protein.

### [Fe-S] cluster reconstitution of SmsCB proteins

All steps were performed under anoxic conditions inside a Jacomex glovebox (<1 ppm oxygen). SmsCB proteins were pretreated under anoxic conditions with 3 mM DTT or TCEP in Tris-HCl 50 mM pH 7.8, NaCl 10 mM. Then, 5 molar excess of ammonium iron(II) sulfate hexahydrate and sodium sulfide were added per nmol of SmsC_2_B_2_. Unbound iron and sulfide were removed by passage on a NAP-25 column (Cytiva). UV-visible spectra (250–750 nm) were recorded on an Avantes Havalight DHS spectrophotometer connected to the glove box by optical fibers to monitor cluster formation. Colorimetric assays were used to measure the iron [43] and sulfur [44] content after reconstitution.

For [Fe-S] cluster reconstitution on SmsC variants, the same procedure was performed on SmsC proteins obtained from recombinant His-tagged SmsC (produced from pVP428, pVP430, pVP487 plasmids) and SmsCB (produced from pVP367 plasmid) proteins in *E. coli*.

A similar procedure was performed for the ^57^FeS reconstitution of the SmsC (12.6 mg) and SmsC_2_B_2_ (9 mg), for Mössbauer spectroscopic studies. To reach 467 µM SmsC_2_ and 350 µM SmsC_2_B_2_, we used concentration Amicon Ultra device. 4 mM of DTT was used in the pretreatment and 4 equivalents of 95% ^57^Fe-enriched Mohr’s salt ((NH_4_)_2_Fe(SO_4_)_2_·6H_2_O) and Na_2_S were used per SmsC_2_B_2_.

The incubation lasted 3 h. Unbound iron and sulfide were removed by passage on a NAP-25 column (Cytiva). A Mössbauer cup in Delrin was immediately filled and frozen in cooled 2-methyl-butane inside the glove box and kept in liquid nitrogen until the measurement. The recorded UV-visible spectrum is shown in S1 Fig for the complex SmsC_2_B_2_ and was similar to that displayed in Fig 1D for SmsC.

### ATPase activity assays

The ATPase activity of purified proteins was measured by an enzyme-coupled spectrophotometric assay [45]. The coupling between ATP hydrolysis and NADH oxidation was achieved by excess of pyruvate kinase (PK) and lactate dehydrogenase (LDH) and by monitoring the decrease of NADH at 340 nm using a Jasco V-730 spectrophotometer. SmsC samples (*n* = 3) were added to 1 ml of 25 mM Hepes buffer (pH 7.6) containing 100 mM KCl, 5 mM MgSO_4_, 5 mM phospho-enol pyruvate, 1 mM NADH, 5 UI of PK, and 10 UI of LDH. Then, 1 mM of ATP was added to initiate the reaction at 25 °C. Specific activities were calculated using the molar extinction coefficient of 6.22 mM^−1^ cm^−1^ for NADH and the SmsC protein concentration determined from its extinction coefficient as previously described. For Kd measurement, a concentration range of SmsC proteins from 0 to 40 µM and SmsC_K45R_ proteins from 0 to 25 µM were incubated with a fixed 400 nM concentration of mantATP*y*S (2′/3′-O-(N-Methyl-anthraniloyl)-adenosine-5′-(γ-thio)-triphosphate, Triethylammonium salt from Jena Bioscience *λ*_max_ 255/355 nm, ɛ 23.3/5.8 L mmol^−1^ cm^−1^ (Tris-HCl pH 7.5), *λ*_exc_ 355 nm, *λ*_em_ 448 nm) [46] in HEPES buffer 25 mM, KCl 100 mM, MgSO_4_ 5 mM, pH 7.6. Then, *λ*_exc_ 355 nm and *λ*_em_ 448 nm were used for mantATP*y*S fluorescence measurements.

Then dissociation constant was calculated using the following equation [*y* = *m*1 * *x*/(*m*2 + *x*)], with *y* being the observed fluorescence signal, *x* the concentration of free ligand, *m*1 the maximum binding signal, and *m*2 the dissociation constant (*K*_d_) [46]. The same *λ*_exc_ 355 nm and *λ*_em_ 448 nm parameters were used for relative fluorescence intensity measurements, with the exception of a scan from 400 to 600 nm to obtain the spectrum of relative fluorescence intensity. For this experiment, a fixed concentration of 50 µM of proteins and 21 µM of mantATP*y*S were used.

### Mössbauer spectroscopy

Mössbauer spectra were recorded at 5.8 K on a strong-field Mössbauer spectrometer equipped with an Oxford Instruments Spectromag 4000 cryostat containing an 8 T split-pair superconducting magnet. The spectrometer was operated in a constant acceleration mode in transmission geometry. Velocity and absorption values were obtained after the classical folding procedure of the crude recorded data (channel number and counts of photons per channel). The error is the square root value of the counts expressed in percentage versus the counts associated with the baseline. The isomer shift is referenced against that of a metallic iron foil at room temperature. The spectra were analyzed with a home-made program and simultaneously simulated [47,48]. Similar simulated spectra can be obtained using the WMOSS Mössbauer Spectral Analysis Software (www.wmoss.org, 2012–2013, Web Research, Edina).

### In vitro [Fe-S] transfer to aconitase

To assess aconitase activation [49], apo-AcnB from *E. coli* (0.5 nmol) was pretreated with DTT. Then, apo-AcnB was desalted using a Microbiospin column (Biorad) and incubated under anoxic conditions in a glove box at 18 °C in aconitase buffer (50 mM Tris-HCl pH 7.6) with 1.74 equivalents of the reconstituted SmsC_2_B_2_ (2.9 Fe and 3.0 S/SmsC_2_B_2_) or 1.70 equivalents reconstituted SmsC_2(K45R)_B_2_ (3.0 Fe and 3.1 S/SmsC_2(K45R)_B_2_) in order to provide 5 Fe and 5 S atoms per AcnB monomer. After 30 min of incubation, aconitase activity was assessed as described [50]. Briefly, AcnB, SmsC_2_B_2_, and SmsC_2(K45R)_B_2_ mixtures were added to 0.6 mM MnCl_2_, 25 mM citrate, 0.5 U isocitric dehydrogenase, 0.25 mM NADP^+^, 50 mM Tris-HCl pH 7.6, in a 100 μl final volume and NADPH formation was monitored at 340 nm by UV-visible absorption spectroscopy. To assess the effect of ATP during [Fe-S] transfer, pre-reduced apo-AcnB (0.5 nmol) was incubated with 1.74 equivalents of reconstituted SmsC_2_B_2_ (2.9 Fe and 3.0 S/ SmsC_2_B_2_) in the presence or absence of 1 mM ATP and 2 mM MgCl_2_. After 1, 2, or 10 min incubation the aconitase activity was measured. The AcnB activity corresponds to the initial velocity of isocitrate production (µM/min). The positive control corresponds to the activity of the chemically reconstituted AcnB (RecAcnB) prepared by incubating apo-AcnB with 5 molar excess of ferrous iron and sulfur for 30 min in the presence of 500 µM DTT (Activity of 76 ± 2 µM/min). The experiment was performed in duplicate.

### X-ray crystallography: Crystallization, data collection, and structure determination

Screenings of crystallization conditions were performed in sitting-drop 96-well Greiner plates at the Crystallography Core Facility of the Institut Pasteur [51]. Crystallization hits were optimized in 24-well plates using the hanging drop method. Colorless monoclinic crystals (space group P2_1_) of the SmsB_2_C_2_ complex grew in wells containing 0.1 M ammonium sulfate, 0.3 M sodium formate, 0.1 M sodium acetate, 3% w/v PGA (Na+ form, LM) and 3% w/v PEG20000 at pH 5.0 in the reservoir under anoxic conditions. Orthorhombic crystals (space group C222_1_) of SmsB_2_C_2_ were obtained in 5% w/v PEG3350, 10% w/v Tacsimate under oxic conditions and soaked in the reservoir solution supplemented with AMP-PNP for 10 min before freezing in liquid nitrogen for diffraction data collection. Crystals of SmsC_2_ in complex with AMP-PN grew in wells containing 0.1 M NaCl, 0.1 M Bicine pH 9, 30% w/v PEG MME 2K.

X-ray diffraction data were collected at beamlines PROXIMA 1 and PROXIMA 2A (Synchrotron SOLEIL, St. Aubin, France) and processed with autoPROC [52]. The crystal structures of the SmsB_2_C_2_ and SmsC were solved by the molecular replacement method with Phaser [53], using trimmed AlphaFold3 [54] models as search probe. The final models were obtained through interactive cycles of manual model building with Coot [55] and reciprocal space refinement with Buster [56].

Atomic coordinates and structure factors have been deposited in the RCSB Protein Data Bank under the accession codes 9H7Y, 9HBL, and 9H7X.

### Cryo-EM sample preparation

SmsC_2_B_2_ samples prepared and sealed under anoxic conditions were quickly vitrified for cryo-EM experiments on previously glow-discharged Quantifoil R0.6/1 Cu-mesh 300 grids, using a Vitrobot Mk. IV (Thermo Fisher Scientific), blotting for 4 seconds at 100% humidity and 22 °C before plunge freezing.

#### Cryo-EM data acquisition, image processing, and model building.

Data acquisition was carried out in a Titan Krios electron microscope equipped with a Falcon 4i direct electron detector with a Selectris X energy filter (Thermo Fisher Scientific). Two datasets were acquired: 10,883 movies with no stage tilt and 3,565 movies with 30° stage tilt. Patch motion correction and CTF estimation were carried out in CryoSPARC v4.5.1 [57], as well as the downstream image processing. Data acquisition parameters and model-building statistics are summarized in Table 5, and cryo-EM data processing workflow is depicted in S5 Fig.

**Table 5:** Cryo-EM data collection, refinement, and validation statistics.

	[4Fe-4S]-bound SmsC_2_B_2_ complex (EMDB-51913) (PDB-9H78)
**Data collection and processing**	
Magnification	200,000
Voltage (kV)	300
Electron exposure (e–/Å^2^)	40
Defocus range (μm)	−0.8 to −1.4
Pixel size (Å)	0.6
Symmetry imposed	–
Initial particle images (no.)	1,009,413
Final particle images (no.)	277,904
Map resolution (Å)	2.8
FSC threshold	0.143
Map resolution range (Å)	2.5–4.0
**Refinement**	
Initial model used (PDB code)	–
Model resolution (Å)	2.64
FSC threshold	0.143
Model resolution range (Å)	2.5–4.0
Map sharpening *B* factor (Å^2^)	−71.56654
Model composition	
Non-hydrogen atoms	8,272
Protein residues	1,056
Ligands	1
*B* factors (Å^2^)	
Protein	0.44/142.90/46.78
Ligand	20.32/42.63/32.95
R.m.s. deviations	
Bond lengths (Å)	0.013 (0)
Bond angles (°)	1.958 (31)
Validation	
MolProbity score	0.56
Clashscore	0.06
Poor rotamers (%)	1.10
Ramachandran plot	
Favored (%)	98.09
Allowed (%)	1.72
Disallowed (%)	0.19

Our crystal structure of SmsC_2_B_2_ was used as an initial model for model building of the [Fe-S] cluster-loaded SmsC_2_B_2_ cryo-EM structure. Model building and refinement were carried out in Coot v0.9.8 [55] and Phenix v1.20.1-4487-000 [58]. Structure model and map have been deposited in the RCSB Protein Data Bank and the EMDB under the accession code 9H78 and EMD-51913, respectively.

## Supporting information

S1 Fig Mössbauer analysis on SmsC_2_B_2_ bound to [Fe-S]. **(a)** UV-visible spectrum of SmsC_2_B_2_ reconstituted for Mössbauer analysis**. (b)** 6 K Mössbauer spectra (black vertical bars) of SmsC_2_B_2_ (350 µM, 3.6 Fe and 3.4 S/ SmsC_2_B_2_) recorded using a 0.06 T (A) and a 7 T (B) external magnetic field applied parallel to the γ-beam. The simulations assuming two iron sites in a 3:1 ratio are overlaid as thick red solid lines and the major and minor contributions are displayed above as blue and mauve thin solid lines, respectively. The nuclear parameters are: major component (75%): *δ*_*1*_ = 0.42 ± 0.01 mm s^−1^, *∆E*_*Q,1*_ = 1.23 ± 0.05 mm s^−1^ and *η*_*1*_ = 0.8 ± 0.1; minor component (25%): *δ*_*2*_ = 0.40 ± 0.01 mm s^−1^, *∆E*_*Q,2*_ = 0.83 ± 0.05 mm s^−1^ and *η*_*2*_ = 0.2 ± 0.1. The data underlying this figure can be found in S1 Fig and S5 Data.
(TIFF)

S2 Fig Mössbauer analysis on SmsC bound to [Fe-S]. **(a)** UV-visible spectrum of SmsC reconstituted for Mössbauer analysis**. (b)** 6 K Mössbauer spectra (black vertical bars) of SmsC (935 µM, 1.7 Fe and 1.6 S/ SmsC) recorded using a 0.06 T (A) and a 7 T (B) external magnetic field applied parallel to the γ-beam. The blue solid line was calculated assuming a diamagnetic Fe site that accounts for 90 ± 5% of the total iron content. The nuclear parameters are: isomer shift = 0.45 ± 0.01 mm s^−1^, quadrupole splitting *ΔE*_*Q*_ = 1.11 ± 0.05 mm s^−1^ and EFG rhombicity = 0.6 ± 0.1. They are strongly reminiscent of those determined for cysteine-coordinated [4Fe-4S]^2+^ clusters. The remaining area (≈10% of the iron content) may correspond to high-spin Fe^II^ impurities. The data underlying this figure can be found in S2 Fig and S6 Data.
(TIFF)

S3 Fig SmsC_K45R_ binds [Fe-S] cluster. UV-Vis absorption spectrum of SmsC_K45R_. SmsC_K45R_ (42 μM) was incubated with 5 equivalents of Fe^2+^/SmsC_(K45R)2_, 5 equivalents of Na_2_S/SmsC_(K45R)2_ and 3 mM DTT. The data underlying this figure can be found in S3 Fig and S7 Data.
(TIFF)

S4 Fig L-Cysteine is not a source of sulfur for the SmsCB complex. Spot test assay for growth indifferent culture dilutions of *Escherichia coli. ΔiscUAΔsuf* MEV carrying the empty pBAD vector (lane 1), the pBAD vector carrying the *E. coli sufABCSDE* operon (lane 2), and the pBAD vector carrying the *Methanocaldococcus jannaschii smsCB operon* (lane 3). Medium was LB supplemented with 0.2% arabinose and different concentrations of l-Cysteine (0.1, 0.5, and 1 mM) as indicated, in oxic or anoxic conditions.
(TIFF)

S5 Fig Cryo-EM data processing workflow. (TIFF)

S1 File Raw Image. **SDS-PAGE as analyzed prior to annotation (see legend** Fig 1a **for details).**
(PDF)

S1 Data Raw Data corresponding to Fig. 1a-1d. Spectroscopy analysis of SmsC_2_B_2_ complex. (ZIP)

S2 Data Raw Data corresponding to Fig. 4ab. Residues C219, C239, and C242 of SmsC act as ligands of the [Fe-S] cluster. (ZIP)

S3 Data Raw Data corresponding to Fig. 5a-5d. Role of ATP binding/hydrolysis in SmsCB. (ZIP)

S4 Data Raw Data corresponding to Fig. 6a-6c. ATP and [Fe-S] cluster binding are mutually exclusive.
(ZIP)

S5 Data Raw Data corresponding to S1 Fig ab. Mössbauer analysis on SmsC_2_B_2_ bound to [Fe-S]. (XLSX)

S6 Data Raw Data corresponding to S2 Fig ab. Mössbauer analysis on SmsC bound to [Fe-S]. (XLSX)

S7 Data Raw Data corresponding to S3 Fig. SmsC_K45R_ binds [Fe-S] cluster. (CSV)

## Abbreviations

AMP-PCP: adenosine-5′-[(β,γ)-methyleno]triphosphate
cryo-EM: cryogenic electron microscopy
GOE: Great Oxidation Event
ISC: Iron-Sulfur Cluster
LB: Luria–Bertani broth
LUCA: Last Universal Common Ancestor
MIS: Minimal ISC System
MVA: mevalonate
NIF: Nitrogen Fixation
SMS: Suf Minimal System
SUF: Sulfur Mobilization
