# Supplementary figures and images for: Ancestral [Fe-S] biogenesis system SMS has a unique mechanism of cluster assembly and sulfur utilization
Source: PLoS Biol. 2025 Jun 25;23(6):e3003223. doi: 10.1371/journal.pbio.3003223 (PMC12192291; doi:10.1371/journal.pbio.3003223)

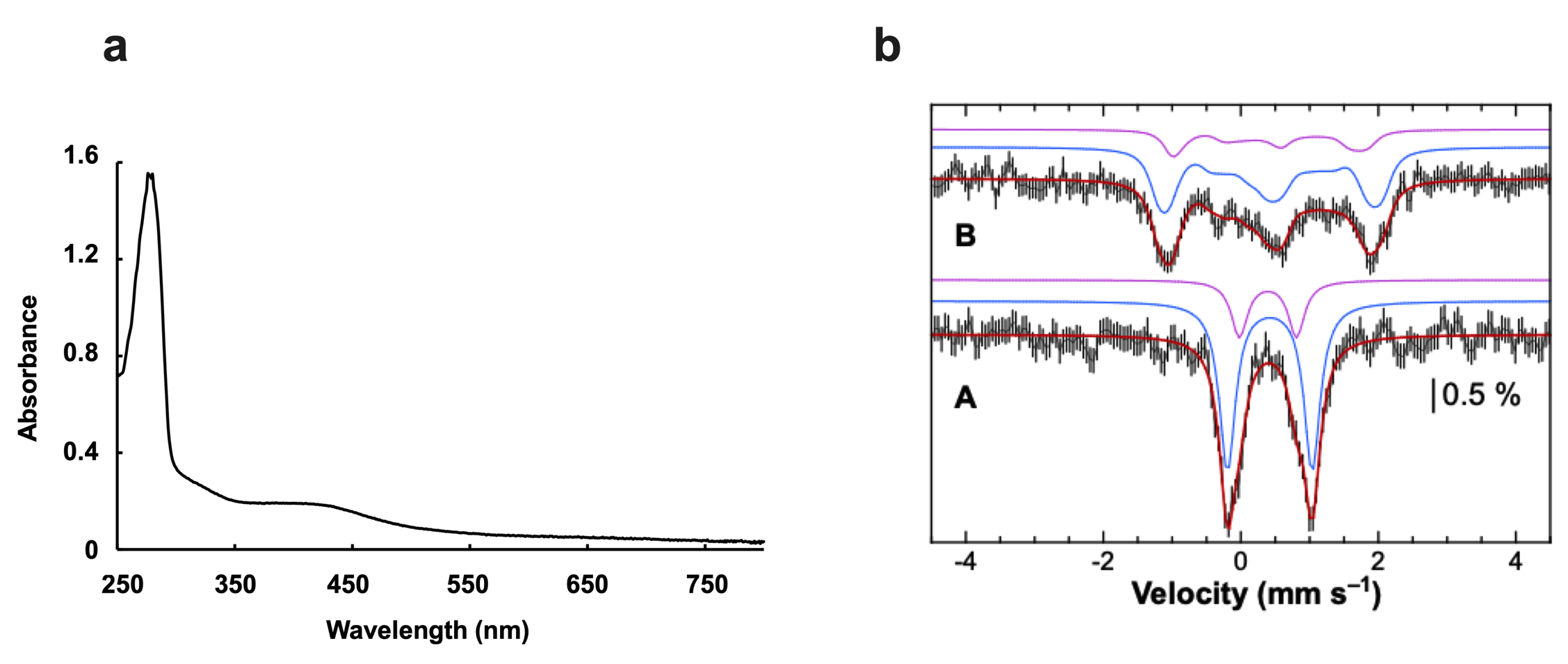

Supplement: S1 Fig — (a) UV-visible spectrum of SmsC2B2 reconstituted for Mössbauer analysis. (b) 6 K Mössbauer spectra (black vertical bars) of SmsC2B2 (350 µM, 3.6 Fe and 3.4 S/ SmsC2B2) recorded using a 0.06 T (A) and a 7 T (B) external magnetic field applied parallel to the γ-beam. The simulations assuming two iron sites in a 3:1 ratio are overlaid as thick red solid lines and the major and minor contributions are displayed above as blue and mauve thin solid lines, respectively. The nuclear parameters are: major component (75%): δ1 = 0.42 ± 0.01 mm s−1, ∆EQ,1 = 1.23 ± 0.05 mm s−1 and η1 = 0.8 ± 0.1; minor component (25%): δ2 = 0.40 ± 0.01 mm s−1, ∆EQ,2 = 0.83 ± 0.05 mm s−1 and η2 = 0.2 ± 0.1. The data underlying this figure can be found in S1 Fig and S5 Data. (TIFF) [file pbio.3003223.s001.tiff]

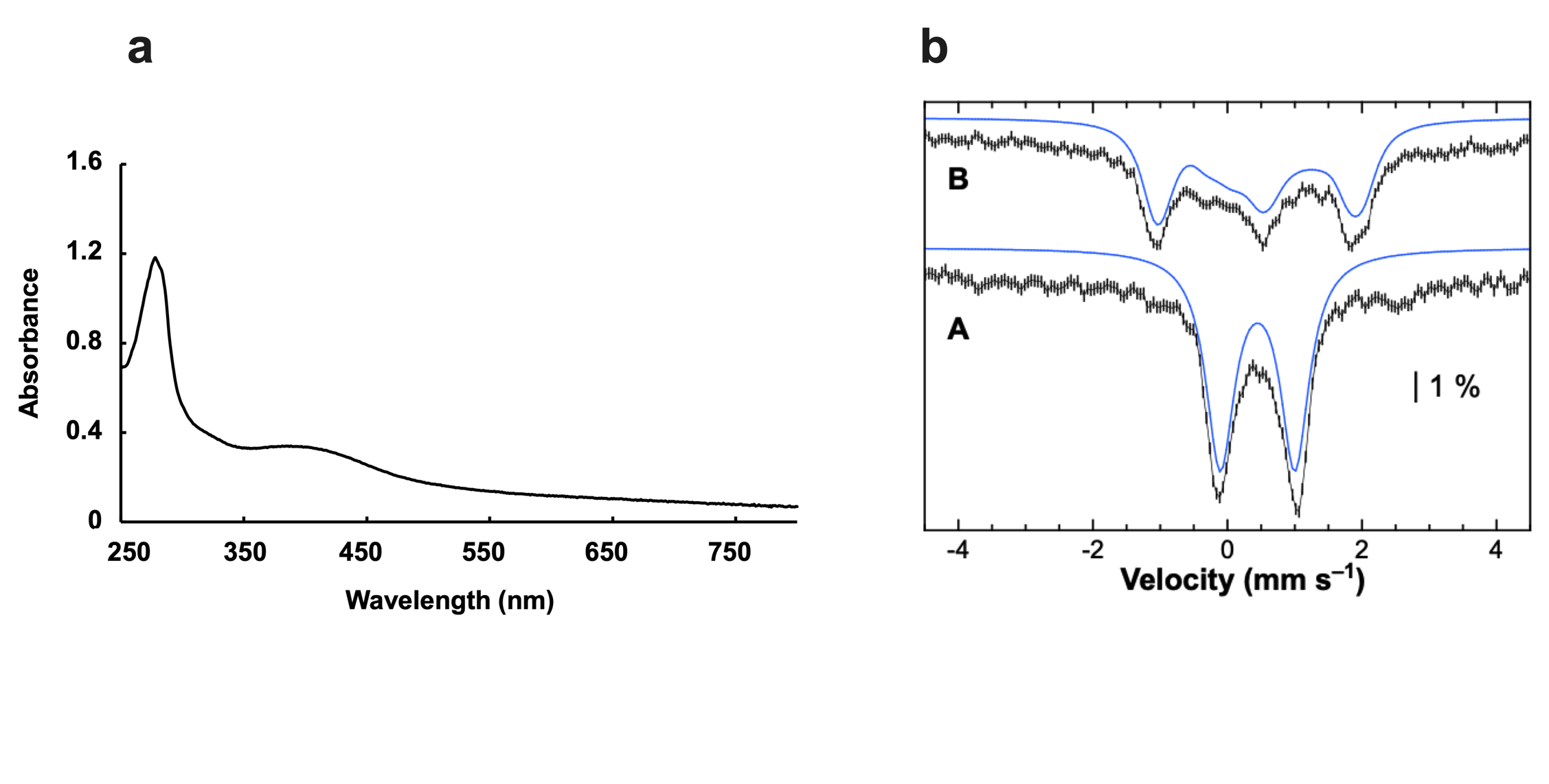

Supplement: S2 Fig — (a) UV-visible spectrum of SmsC reconstituted for Mössbauer analysis. (b) 6 K Mössbauer spectra (black vertical bars) of SmsC (935 µM, 1.7 Fe and 1.6 S/ SmsC) recorded using a 0.06 T (A) and a 7 T (B) external magnetic field applied parallel to the γ-beam. The blue solid line was calculated assuming a diamagnetic Fe site that accounts for 90 ± 5% of the total iron content. The nuclear parameters are: isomer shift = 0.45 ± 0.01 mm s−1, quadrupole splitting ΔEQ = 1.11 ± 0.05 mm s−1 and EFG rhombicity = 0.6 ± 0.1. They are strongly reminiscent of those determined for cysteine-coordinated [4Fe-4S]2+ clusters. The remaining area (≈10% of the iron content) may correspond to high-spin FeII impurities. The data underlying this figure can be found in S2 Fig and S6 Data. (TIFF) [file pbio.3003223.s002.tiff]

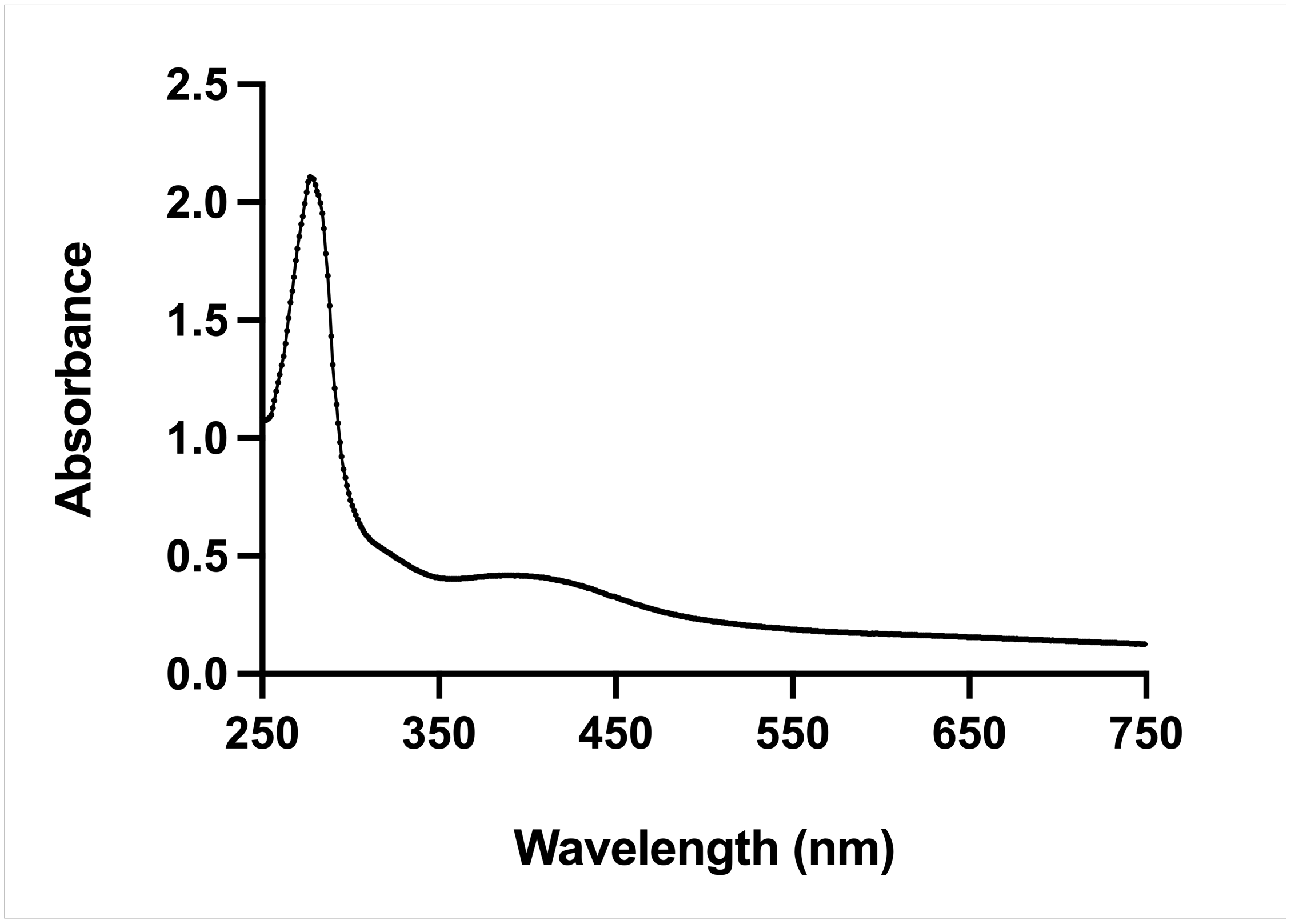

Supplement: S3 Fig — UV-Vis absorption spectrum of SmsCK45R. SmsCK45R (42 μM) was incubated with 5 equivalents of Fe2+/SmsC(K45R)2, 5 equivalents of Na2S/SmsC(K45R)2 and 3 mM DTT. The data underlying this figure can be found in S3 Fig and S7 Data. (TIFF) [file pbio.3003223.s003.tiff]

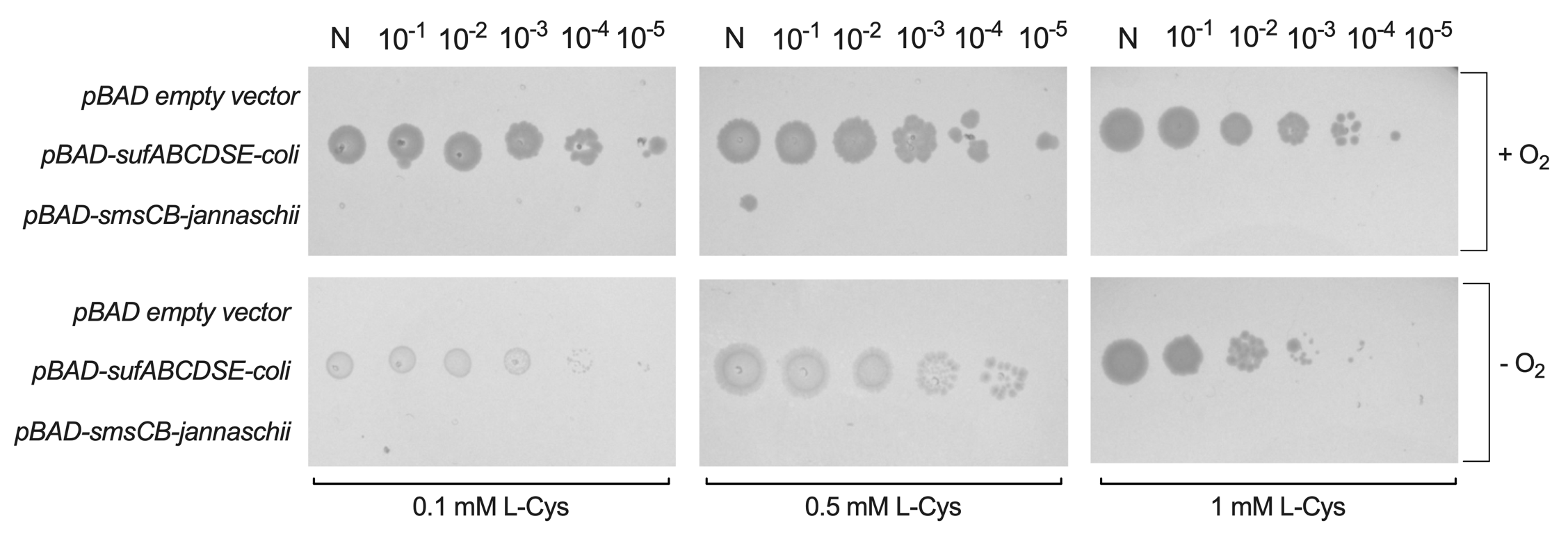

Supplement: S4 Fig — Spot test assay for growth indifferent culture dilutions of Escherichia coli. ΔiscUAΔsuf MEV carrying the empty pBAD vector (lane 1), the pBAD vector carrying the E. coli sufABCSDE operon (lane 2), and the pBAD vector carrying the Methanocaldococcus jannaschii smsCB operon (lane 3). Medium was LB supplemented with 0.2% arabinose and different concentrations of l-Cysteine (0.1, 0.5, and 1 mM) as indicated, in oxic or anoxic conditions. (TIFF) [file pbio.3003223.s004.tiff]

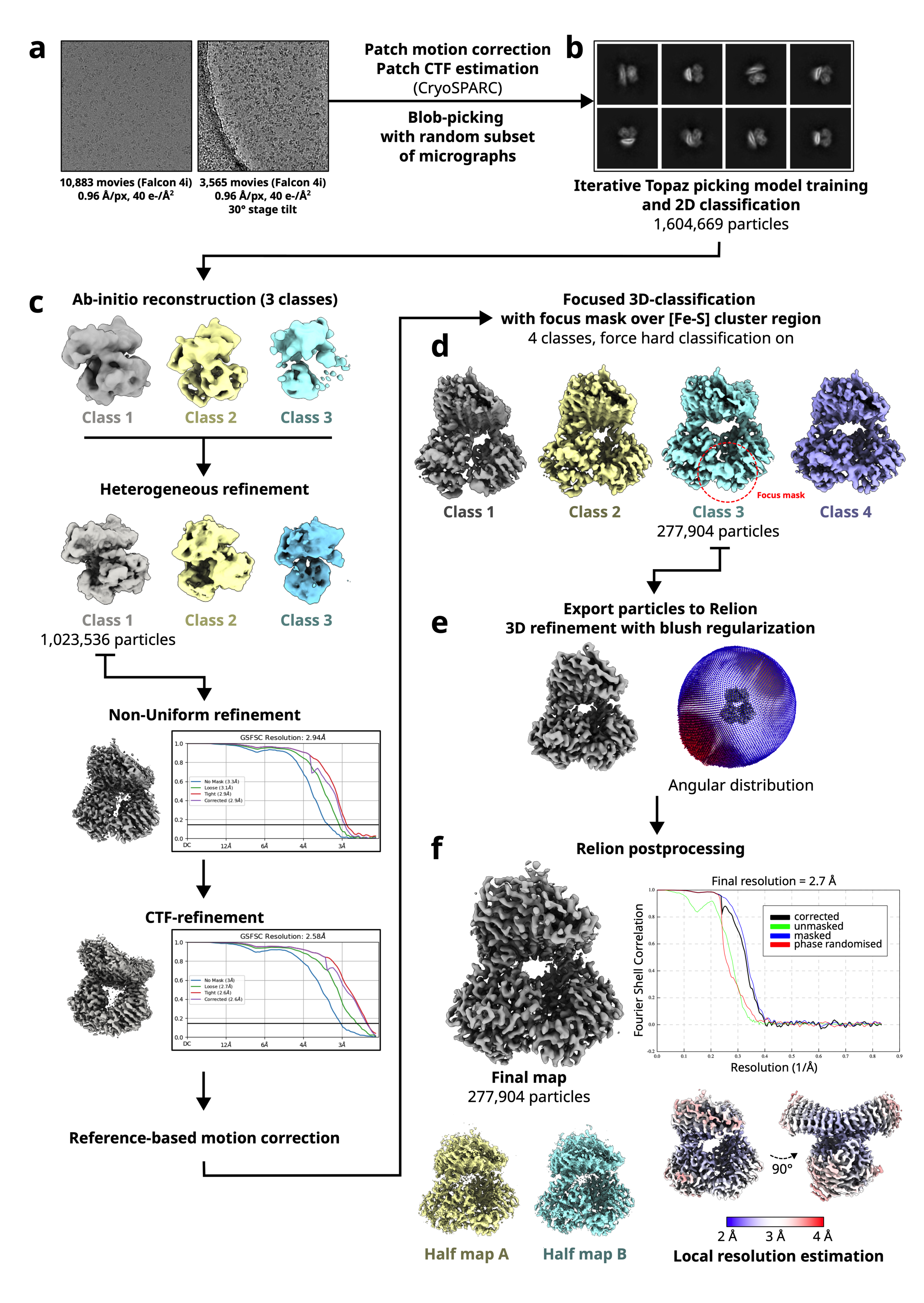

Supplement: S5 Fig — (TIFF) [file pbio.3003223.s005.tiff]

S1\_raw\_images

(a) raw

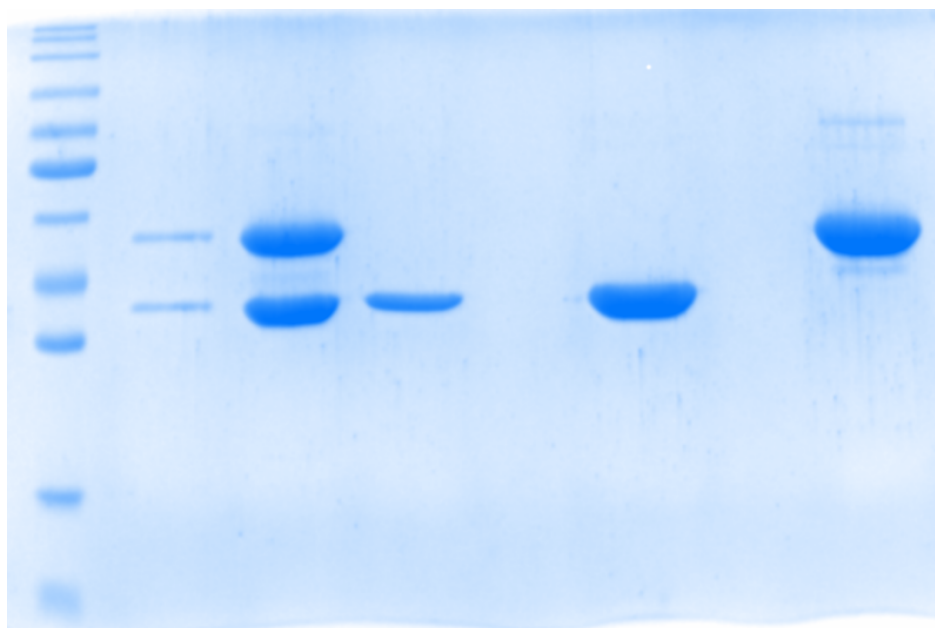

(b) annotated

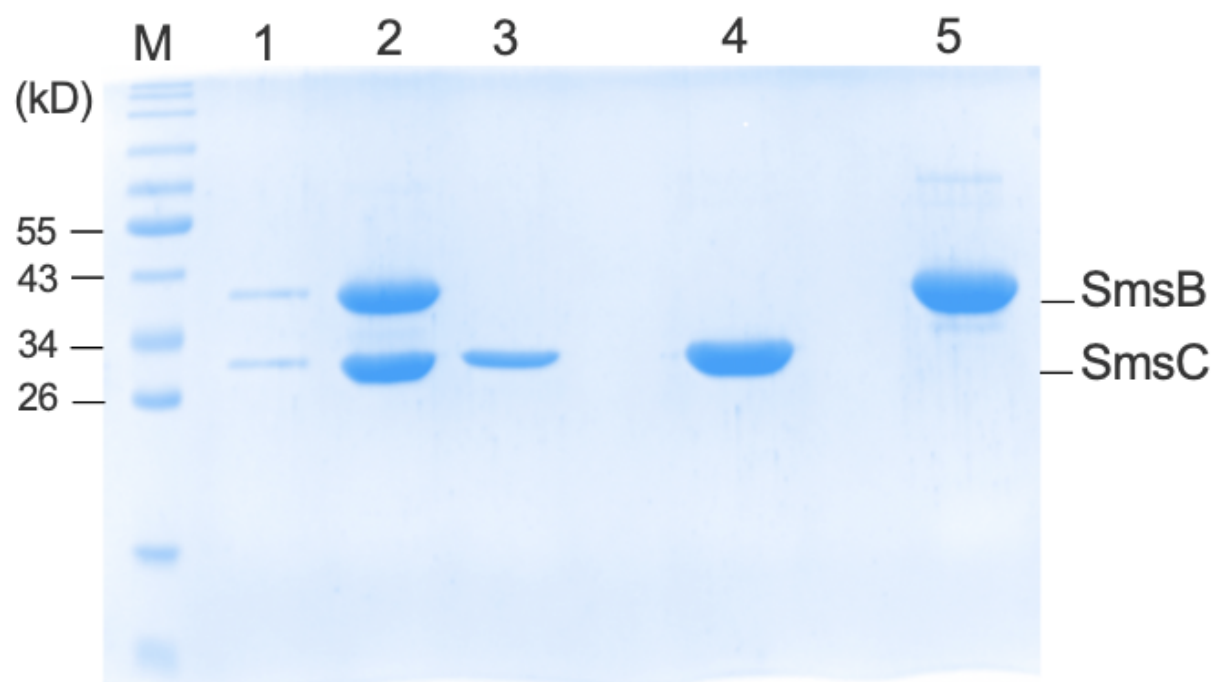

Supplement: S1 File — SDS-PAGE as analyzed prior to annotation (see legend Fig 1a for details). (PDF) [file pbio.3003223.s006.pdf]
